# Global histone protein surface accessibility in yeast indicates a uniformly loosely packed genome with canonical nucleosomes

**DOI:** 10.1186/s13072-020-00381-5

**Published:** 2021-01-11

**Authors:** Luke T. Marr, Josefina Ocampo, David J. Clark, Jeffrey J. Hayes

**Affiliations:** 1 Department of Biochemistry and Biophysics, University of Rochester Medical Center, Rochester, NY 14642 USA; 2 Instituto de Investigaciones en Ingeniería Genética y Biología Molecular “Dr. Héctor N. Torres” (INGEBI-CONICET), C1428ADN Buenos Aires, Argentina; 3 Division of Developmental Biology, Eunice Kennedy Shriver National Institute of Child Health and Human Development, Bethesda, MD 20892 USA

**Keywords:** Subnucleosome, Chromatin structure, Transcription, Remodelers

## Abstract

**Background:**

The vast majority of methods available to characterize genome-wide chromatin structure exploit differences in DNA accessibility to nucleases or chemical crosslinking. We developed a novel method to gauge genome-wide accessibility of histone protein surfaces within nucleosomes by assessing reactivity of engineered cysteine residues with a thiol-specific reagent, biotin-maleimide (BM).

**Results:**

Yeast nuclei were obtained from cells expressing the histone mutant H2B S116C, in which a cysteine resides near the center of the external flat protein surface of the nucleosome. BM modification revealed that nucleosomes are generally equivalently accessible throughout the *S. cerevisiae* genome, including heterochromatic regions, suggesting limited, higher-order chromatin structures in which this surface is obstructed by tight nucleosome packing. However, we find that nucleosomes within 500 bp of transcription start sites exhibit the greatest range of accessibility, which correlates with the density of chromatin remodelers. Interestingly, accessibility is not well correlated with RNA polymerase density and thus the level of gene expression. We also investigated the accessibility of cysteine mutations designed to detect exposure of histone surfaces internal to the nucleosome thought to be accessible in actively transcribed genes: H3 102, is at the H2A–H2B dimer/H3–H4 tetramer interface, and H3 A110C, resides at the H3–H3 interface. However, in contrast to the external surface site, we find that neither of these internal sites were found to be appreciably exposed.

**Conclusions:**

Overall, our finding that nucleosomes surfaces within *S. cerevisiae* chromatin are equivalently accessible genome-wide is consistent with a globally uncompacted chromatin structure lacking substantial higher-order organization. However, we find modest differences in accessibility that correlate with chromatin remodelers but not transcription, suggesting chromatin poised for transcription is more accessible than actively transcribed or intergenic regions. In contrast, we find that two internal sites remain inaccessible, suggesting that such non-canonical nucleosome species generated during transcription are rapidly and efficiently converted to canonical nucleosome structure and thus not widely present in native chromatin.

## Background

The vast majority of eukaryotic genomic DNA is assembled into nucleosomes, the basic repeating subunit of chromatin. In solutions containing physiological ionic strengths long chains of nucleosomes condense into higher order chromatin structures, facilitated by inter-nucleosomal interactions mediated by the core histone ‘tail’ domains [1–3]. While the exact arrangement of nucleosomes within such structures is in debate, most models for higher order structures involve packing and stacking of the disk-shaped nucleosomes along their flat surfaces [2, 4, 5]. Indeed individual nucleosome core particles at high concentrations, and osmotic pressures spontaneously stack in columnar structures of various configurations dependent on ionic conditions [6–12]. Nucleosome–nucleosome interactions are stabilized by linker histones, and may occur between nucleosomes in the same locality or between nucleosomes separated by large distances [2, 13].

Numerous non-histone chromatin factors govern the accessibility of genomic DNA within chromatin to regulate crucial biological processes, including gene expression. Some of these factors act by covalently adding or removing posttranslational modifications such as acetylation and phosphorylation on the core histones which alter electrostatic interactions that directly or indirectly alter chromatin structure [14–18]. Alternatively, such ‘marks’ can serve as binding sites for ancillary factors and activities such as ATP-dependent chromatin remodeling complexes and other architectural proteins that act to either enhance compaction or opening of the chromatin [17, 19–24]. The culmination of these factors results in the activation of specific genes and commensurate decompaction of chromatin and increased accessibility of genomic DNA [25–27].

Efforts to map accessible, open regions of chromatin associated with active genes have relied on determining locations of differential accessibility of genomic DNA to nucleases such as DNase I, micrococcal nuclease, or chemical reagents. For example, DNA regions hypersensitive to DNase I were found to indicate open chromatin regions associated with active genes [27]. Formaldehyde-assisted isolation of regulatory elements (FAIRE) also identifies open, histone-depleted DNA regions in chromatin, which are predominantly associated with promoters and enhancers [28]. More recently, ATAC-Seq has been used to identify accessible DNA regions in chromatin delimited by nucleosomes and other chromatin binding proteins [29, 30]. While the commonly used micrococcal nuclease (MNase) efficiently excises nucleosome core particles, allowing detailed mapping of nucleosome occupancies, [31, 32], recent studies have shown that nucleosomes exhibit distinct rates of appearance and disappearance during MNase digestion, due to regional differences in DNA accessibility reflective of more open or closed global chromatin structures [33–35]. While much has been learned from assays gauging DNA accessibility, there are no techniques to assess how accessibility of the histone proteins varies with respect to genomic elements. Moreover, recent data indicate that accessibility to DNA probes does not strictly correlate with gene activity [35]. Therefore, an alternative measure of chromatin accessibility might provide further insight into how chromatin structure is altered to facilitate biological processes.

We previously described a method to probe the accessibility of surfaces of the core histones in the chromatin of *S. cerevisiae* nuclei. This method involves installation of a rationally selected cysteine substitution within a core histone and limited reaction with the thiol-specific reagent biotin-maleimide to determine the genome-wide accessibility of selected external and internal histone surfaces in nucleosomes. Measurements of histone surface accessibility provide insights complementary to chromatin accessibility derived from nucleases, such as MNase-Seq [36]. Here we monitor the accessibility of H2B S116C, located on an external surface of the nucleosome, the exposure of which is expected to be modulated through inter-nucleosomal interactions. Additionally, we probe the accessibility of two sites within the nucleosome core, H3 S102C and H3 A110C to survey generation of sub-nucleosomal structures in which the H3–H3 or the H3/H4–H2A/H2B interfaces are exposed, respectively. While we observe some differential accessibility for the external surface site, we find this site to be, in general, widely accessible throughout the *S. cerevisiae* genome. In stark contrast, we find little evidence of noncanonical nucleosome structures with exposed internal surfaces.

## Materials and methods

### Yeast strains

Generation of the H2B S116C mutant strain has previously been reported [36]. Briefly, a yeast strain (YDC 417) with one functional *HTA–HTB* locus (*HTA2–HTB2* deleted) is transformed with an integration plasmid bearing the *HTA1–HTB1* locus. The mutation of interest is made via site-directed mutagenesis. In addition to the *HTA1–HTB1* locus, the plasmid also contains a functional copy of *HIS3* which permits transformants to grow in histidine deficient media. Confirmation of proper integration and maintenance of mutation was performed by isolating the genomic DNA and sequencing the region of interest. For the H3 experiments, we used ROY1281 (MATalpha *lys2 trp1 his3 leu2 ura3 hhf1–ht1·::LEU2 hhf2–hht2·HIS3 pRO149 HHF1 HHT1 HTA1 HTB1* [37, 38]. H3 mutations are introduced onto plasmid p368 derived from pRS317 which contains a functional *LYS2* gene [39]. As for H2B S116C, H3 mutations are introduced via Q5 site-directed mutagenesis (NEB E50554S) and sequence confirmed. However, the p368 plasmids are introduced into the strain through plasmid-shuffling rather than integration into the genomic DNA. The p368 plasmid is introduced to ROY1281 cells using the lithium acetate method and plated onto SC-LYS media to screen for transformants [40]. To obtain a strain containing only the p368 plasmid, transformants were inoculated and grown in SC-LYS medium and subsequently plated on SC-LYS with 5-FOA (0.1%) to select for colonies which had exchanged p368 for pRO149 as this plasmid contains a functional *URA3* whose presence will prevent growth on 5-FOA.

### Preparation of biotin-maleimide modified chromatin

Nuclei from control and cells expressing histone mutants were prepared as in [41]. Briefly, yeast cultures were grown to mid-log phase (OD_600_ 0.5–0.8), treated with 6000 units of lyticase (Sigma Aldrich L2524), nuclei isolated with a Ficoll 400 cushion, then resuspended in MNase digestion buffer lacking β-mercapoethanol (BME) and CaCl_2_. Nuclei were modified with biotin-maleimide (BM, ThermoFisher Scientific 21901BID) as described in [36] with minor changes to account for modification times. BM was added to nuclei such that the final concentration was 100 µM and mixed by pipetting up and down 10×. Following mixing, 800 µL (1/3 of the reaction) was removed after 10 s, 1 min, and 10 min of reaction, and transferred to a fresh tube containing 35 µL of 140 mM β-mercaptoethanol (BME, Sigma Aldrich M6250, final concentration 5 mM) to stop the reaction. Seven µL of 62.5 mM CaCl_2_ was then added to final concentration of ~ 0.5 mM and the samples split into two aliquots for digestion with either 480 or 960 units of MNase (Worthington Biochemical LS004798) at room temperature for 3 min. The amount of enzyme was empirically determined to yield predominantly mononucleosomes. Digestion was stopped by the addition of 100 µL MNase stop solution (5 mM EDTA, 5 mM EGTA, 0.05% NP-40) and held on ice for 2 min. Samples are then treated as in [41] with 100 µL of the final digested chromatin being used for analysis of DNA fragment sizes generated from the digestion, and the remaining 400 µL of digested, BM modified chromatin stored at − 80 °C until affinity purification.

### Affinity purification of biotin-maleimide modified nucleosomes

A detailed protocol has previously been reported [36]. Briefly, 200 µL of streptavidin-agarose (Solulink N-1000-002) resin is washed once with 1× PBS pH 7.4 and then 3× with 500 µL of 1× PBS pH 7.4 supplemented with 5 mg/mL BSA (NEB B9000S). MNase digested chromatin (200 µL) is added to streptavidin-agarose and brought to a 1 mL volume with AP binding buffer (10 mM Tris pH 8.0, 1 mM EGTA, 0.05% NP-40, 150 mM NaCl, protease inhibitors (Sigma Aldrich 11873580001). Chromatin is incubated with streptavidin-agarose for 30 min at 4 °C and then washed once with AP binding buffer and twice with AP binding buffer supplemented with 600 mM NaCl. DNA from affinity purified nucleosomes is recovered with two 10 min treatments with elution buffer 1 (1% SDS, 140 mM NaCl, 50 mM Tris pH 8.0, 10 mM EDTA) and elution buffer 2 (0.67% SDS, 140 mM NaCl, 50 mM Tris pH 8.0, 10 mM EDTA).

### Library preparation of input and affinity purified DNAs

Library preparation was carried out with NEBNext® Ultra™ DNA Library Prep Kit for Illumina® (New England BioLabs E7370) and NEBNext® Mutiplex Oligos for Illumina® (New England BioLabs E7335). In addition to yeast input and AP DNAs, we added 1 ng or 10 ng of mouse nucleosome core particle DNA to each sample prior to DNA repair step. This allowed us to standardize fold-enrichment in our experiments by analyzing the ratio of yeast reads to mouse reads between input and AP. A size selection step was not used in the library preparation, and it was found that 5 µL of the adaptor ligated DNA coupled with ten cycles of the PCR conditions yielded sufficient material for sequencing.

### Data analysis of affinity purified and input DNAs

Fastq files were aligned to reference genomes (SacCer2 or mm10) using the default parameters of Bowtie2 to generate SAM files [42]. SAMtools was then used to generate BAM, sortedBAM and indexed BAM files using the default parameters [43]. BAM files were then used to generate bigwig files for individual input and AP files as well as AP/input files using deepTools bamCoverage and bamCompare [44]. For individual input and AP files using bamCoverage, the following arguments were used --binSize 10 --effectiveGenomeSize 12,100,000 --normalizeUsing RPKM --extendReads --ignoreDuplicates --minFragmentLength 135 --maxFragmentLength 165. For AP/input using bamCompare, the same arguments were used as for bamCoverage with the addition of --operation ratio. To map nucleosome fragments and determine accessibility, we employed computeMatrix and plotHeatmap from deepTools. Gene lists used to generate figures in this manuscript are cited in “Results” section. The determination of the amount of yeast DNA and ultimately the percentage of biotin-maleimide modified nucleosomes was accomplished by the inclusion of a known quantity of mouse NCP DNA, typically 1 ng, prior to DNA library preparation. The ratio of aligned yeast DNA reads with respect to mouse DNA reads was multiplied by the amount of spiked in mouse DNA to determine the amount of yeast DNA. In the case of the AP samples we applied a correction to account for the difference in MNase digested chromatin used to generate input and AP samples, as described in previous section, and that 10% of the input DNA compared to 100% of the AP DNA was used for library preparation.  The amount of AP DNA is then divided by the amount of input DNA for each WT and H2B S116C time point to determine the percentage of H2B S116C modified nucleosomes at each time point. The values for WT were deemed to be background and therefore the average WT value was subtracted from each H2B S116C time point to determine the true percentage of H2B S116C modified nucleosomes (‘corrected modified nucleosomes’ in Additional file 1: Table S2).

## Results

### Distinct nucleosome surface accessibility in genes

**Fig. 1:**
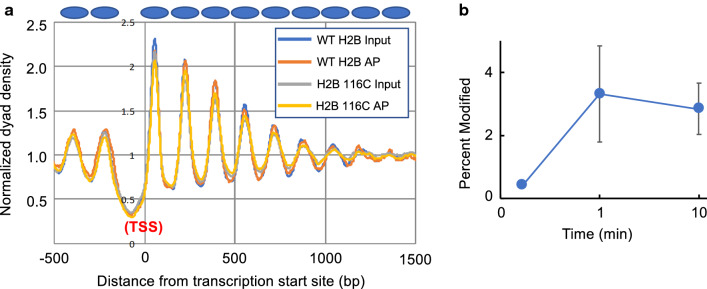
**a** Nucleosome phasing plots for input and affinity purified (AP) samples from WT and H2B S116C cells, as indicated. Plots were aligned to the transcription start sites (TSS) of yeast genes and normalized to total reads for each data set. **b** Percent nucleosomes modified by biotin maleimide. Modification was carried out for 10 s, 1 and 10 min, as described in the text. Values were derived from data in Additional file 1: Table S2 as described in “Materials and methods”

We first sought to determine the extent to which the accessibility of an external histone protein surface on nucleosomes varies genome-wide among genes in yeast cells. We employed a new method in which modifiability of an engineered cysteine residue is probed with the thiol-specific reagent biotin maleimide (BM) [36]. The histone mutant H2B S116C places a cysteine exposed to solvent near the center of the flat protein surface of the nucleosome [36]. This surface is occluded upon condensation of oligonucleosome arrays [4, 45, 46], and upon nucleosome–nucleosome stacking in crystals and condensed liquid crystalline preparations of nucleosome core particles [6, 7, 11, 18, 47]. Indeed, we find that a cysteine at the corresponding site in *Xenopus* H2B (S112C) is more accessible in expanded oligonucleosome arrays than condensed arrays (results not shown). Freshly prepared nuclei from yeast cells expressing H2B S116C as the sole source of H2B were incubated with BM, digested with micrococcal nuclease, and BM modified nucleosomes affinity-purified (AP) and sequenced [36] (Additional file 1: Table S1). We first confirmed the expected nucleosome phasing (Fig. 1a). Phasing plots indicated virtually identical average nucleosome positioning/spacing over Pol II genes both WT and H2B S116C samples (Fig. 1a). We also examined the extent of nucleosome modification as a function of time as we wished to avoid saturating nucleosome modification sites to ensure distinction between nucleosomes with differentially accessible surfaces. Accordingly, we found about 0.4% of nucleosomes were modified when nuclei where incubated with BM for 10 s, but the reaction reached an apparent limit of 3.1 ± 1.4% of all nucleosomes modified at 1 and 10 min of modification, likely due to exhaustion of reagent upon reaction with the excess cysteine targets available in the nucleus (Fig. 1b and Additional file 1: Table S2).

**Fig. 2:**
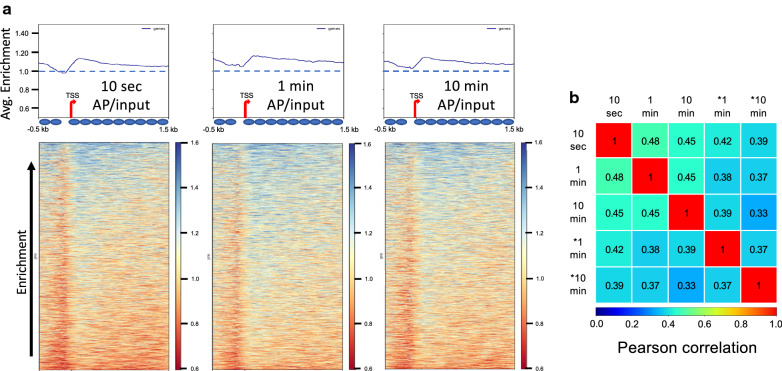
H2A S116C accessibilities across *S. cerevisiae* genes. **a** Heat maps of yeast Pol II genes sorted in descending order according to H2B S116C AP/input scores averaged over the region − 0.5 to + 1.5 kb around the TSS. BM modification was carried out for 10 s, and 1 and 10 min, as indicated. Top, plot of cumulative AP/input score vs position relative to TSS for all genes in the heat map (bottom). The average location of individual nucleosomes determined from phasing profiles (Fig. 1) are indicated by the ovals below the plot. **b** Pearson correlations between the three experiments shown and replicate experiments for the 1 and 10-min modifications, indicated by asterisks

We next used the normalized AP and matched input data directly to determine nucleosome accessibility for the above data and replicate experiments (Additional file 1: Table S3). We aligned the genomic AP/input profiles according to transcription start sites (TSS) for 4166 yeast Pol II genes within a region 0.5 kb upstream to 1.5 kb downstream of the TSS (referred to as ‘whole gene’ analysis) and plotted the data averaged for all genes for each time point (Fig. 2a, top). Thus, an accessible or ‘enriched’ nucleosome would exhibit an AP/input score greater than 1, whereas an inaccessible nucleosome would exhibit a score less than 1. The profiles show an average nucleosome enrichment of approximately 1 over the selected 2 kb region encompassing the TSS, including the nucleosome depleted region. However, we observe a small peak in enrichment just downstream of the TSS, which tails off further into the gene, and a slightly lower score across the NDR, likely due to a de-enrichment of non-nucleosomal species in the AP. These results indicate that, in aggregate, Pol II genes exhibit accessibilities that are slightly higher than the genomic average but approximately equivalent through the upstream and downstream region.

**Fig. 3:**
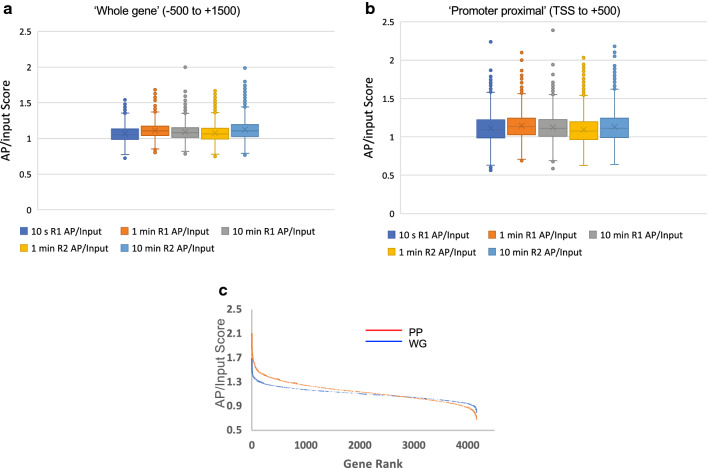
Dynamic range of accessibility scores. Average AP/input scores for each gene were determined for ‘whole gene’ (**a**) and ‘promoter proximal’ (**b**) windows, as indicated, for each determination and plotted in box and whisker format. Shown are data from the 10 s (blue), 1 min (orange), and 10 min (grey) BM modification time points, and 1 min (yellow) and 10 min (light blue) replicate experiments. **c** Distribution of accessibility scores. The average AP/input scores for each gene were determined and plotted according to rank (1–4166) for the ‘whole gene’ − 500 to + 1500 (blue line, ‘WG’) and ‘promoter proximal’ TSS to + 500 (orange line, ‘PP’) windows for the 1 BM modification minute time point

To determine whether yeast genes exhibit differential accessibilities, we sorted genes aligned by TSS according to average AP/input enrichment score for each gene. Comparison of results from the three time points and replicate experiments (Additional file 1: Tables S1–S3) indicates that yeast genes exhibit a range of nucleosome surface accessibilities (Fig. 2a, heatmaps). The average correlation of gene ordering according to AP/input score between time points and independent experiments was moderate but significant (~ 0.40 Pearson correlation, TSS to TTS) (Fig. 2b). We generated box and whisker plots of the average AP/input score for all genes to gauge the range of accessibility scores between each experiment (Fig. 3a). We find that the mean and medians are almost identical within and across experiments (Fig. 3a and Additional file 1: Table S4A). Calculation of the inter-quartile range (IQR) reveals the middle 50% of the AP/input scores are distributed over a relatively narrow range with an average IQR of 0.141. To further understand the data, we plotted average accessibility scores for each data set as a function of gene order (Fig. 3c, blue line). The scores for the middle 80% of genes only cover a 1.3-fold range from 0.96 to 1.25, while the average of the top and bottom 5% of scores (1.38 ± 0.08 and 0.9 ± 0.03, respectively) exhibits a range of ~ 1.5. These findings indicate that most genes, on average over the nucleosomes within the 2 kb region immediately surrounding the TSS, have relatively similarly accessible protein surfaces.

We then focused on the first ~ 500 bp of genes, which exhibited a peak in accessibility compared to more upstream and distal regions, and an apparent greater variability among genes (Fig. 2a, top). We resorted genes according to average AP/input scores based on the promoter proximal 500 bp region of each gene and confirmed a greater dynamic range in this region as compared to the ‘whole gene’ window. The average IQR for the 500 bp promoter proximal region was 0.224, approximately 60% greater than that observed for the ‘whole gene’ analysis (Fig. 3b and Additional file 1: Table S4B). Additionally, we find that the minimum and maximum values span a wider range (0.62–2.12) than that of the ‘whole gene’ (0.77–1.78). Plots of promoter proximal AP/input scores by rank show a greater (vertical) spread in scores; with averaged accessibility scores for the middle 80% of genes covering a 1.7-fold range, from 0.86 to 1.43, while the average of the top and bottom 5% of scores was 1.55 ± 0.12 and 0.81 ± 0.05, respectively, a nearly twofold difference in average accessibility (Fig. 3c, orange line).

**Fig. 4:**
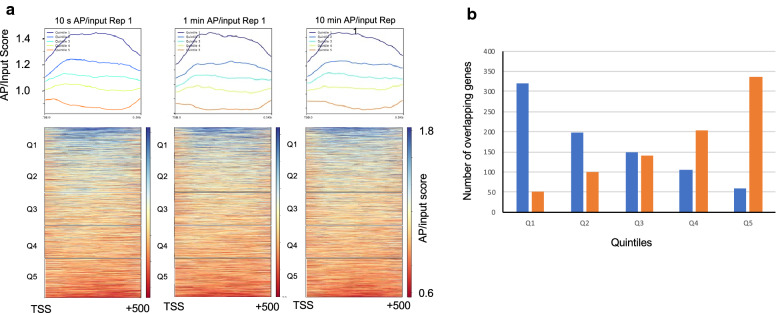
Promoter proximal nucleosomes exhibit relatively larger differences in H2B S116C accessibility relative to the genomic average. **a** Genes were sorted according to AP/input scores averaged over the region TSS to 0.5 kb, then binned into quintiles, with quintile 1 containing genes with the highest AP/input scores and quintile 5 containing the lowest scores. Top, plots of cumulative AP/input scores for each quintile, as shown in the heat map (bottom). **b** Distributions of most and least accessible genes in replicate experiments. Shown is the distribution of genes sorted into quintiles 1 (blue) and 5 (orange) for the 1 min BM modification experiment (Fig. 3a), in quintiles of a replicate experiment

To investigate further, we grouped genes into ranked quintiles (~ 833 genes per quintile) according to average accessibility scores for the promoter proximal (TSS to 0.5 kb) region. For all time points, the average of quintile 1 genes was ~ 1.45 while that for quintile 5 genes was ~ 0.9 (Fig. 4a), indicating nucleosomes within the first 500 bp of genes in quintile 1 are ~ 60% more accessible than those found in quintile 5. In addition, we find that the promoter proximal input nucleosome reads are more similar across all quintiles than total AP reads, which exhibit a much broader distribution across quintiles, indicating discrimination between quintiles is primarily due to differential nucleosome modification and affinity purification (Additional file 1: Figure S1). Comparison of promoter proximal data exhibit similar, moderate levels of correlation among time points of modification and independent experiments, similar to the “whole gene” analysis (Additional file 1: Table S5, “All genes”).

Given that the majority of genes in the center of the distributions exhibit small differences in average accessibility scores, we examined whether genes with more extreme accessibility values were more strongly correlated with each other across data sets. Because the majority of genes differ in accessibility only modestly, we reasoned that this contributed to the moderate Pearson correlation score and that greater Pearson correlation scores would be observed for the most and least accessible genes, Q1 and Q5, respectively. We assessed correlations independently between genes in quintiles 1 and 5 and for quintiles 2–4 for all data sets (Additional file 1: Table S5). Indeed, we find that there is greater correlation amongst quintiles 1 and 5 (average Pearson correlation 0.439) compared to that for all quintiles (0.320) and even more so amongst quintiles 2–4 (average Pearson correlation 0.158). Moreover, a plot of the distribution of genes comprising quintiles 1 and 5 shows that each is found with highest probability in the same quintiles in a replicate experiment (Fig. 4b). Overall, this suggests that while a number of nucleosomes exhibit similar external surface accessibilities, those which exhibit higher and lower scores are not random as these are more strongly correlated with each other across modification time points. This implies that some external nucleosome surfaces in *S. cerevisiae* may in fact be related to nuclear processes.

### Nucleosome surface accessibility is only weakly correlated with RNAPII occupancy

**Fig. 5:**
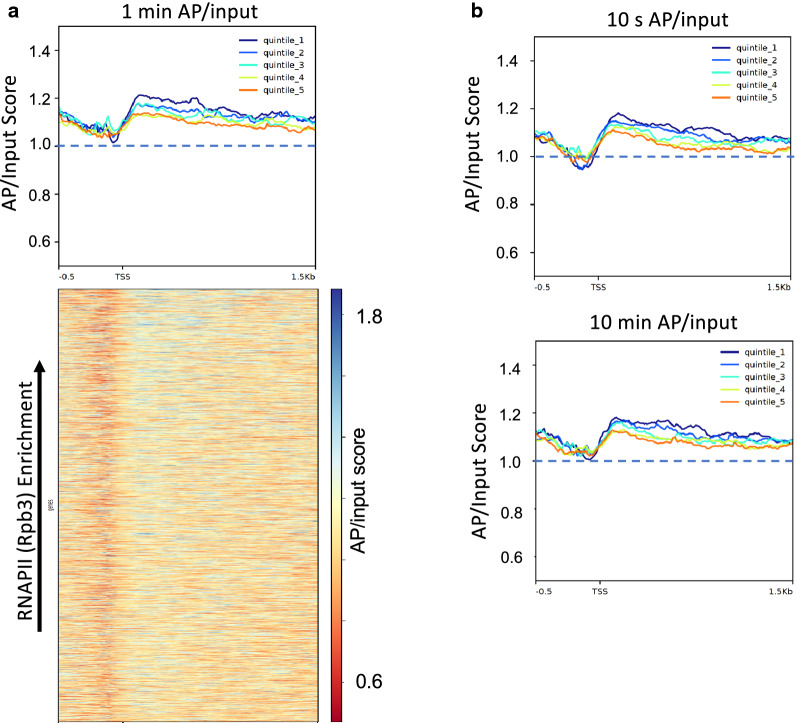
Weak correlation between H2B S116C accessibility and Rpb3 (RNAPII) density. **a** Quintile profiles and heatmap for 1 min AP/input. Genes were sorted according to Rpb3 density and binned into quintiles where quintile 1 contained the genes with the greatest Rpb3 density and quintile 5 the least. AP/input scores were then plotted for genes preserving the Rpb3 ranking in each quintile. **b** Profiles for 10 s and 10 min experiments exhibit similar ordering and minor differences between quintiles as in **a**

Since transcriptionally active chromatin is thought to exist as a more open chromatin structure, we hypothesized that H2B S116C accessibility would positively correlate with active transcription. Genes were sorted into quintiles using ChIP-seq data from an RNA polymerase II subunit, Rpb3 [48]. Quintile 1 contained the genes with the greatest Rpb3 density while quintile 5 contained the genes with the least. We then plotted the AP/input values for these genes to determine whether genes with more Rpb3, and presumably more transcriptionally active, had more accessible external nucleosome surfaces. We found that genes within quintile 1 with the greatest Rpb3 density exhibit only ~ 10% greater accessibility scores on average compared to quintile 5, with the least Rpb3 density, regardless of modification time (Fig. 5a, b). Despite our finding that accessibility increased with Rpb3 density, the absolute difference in average accessibility between each successive quintiles was extremely small (Fig. 5a, b). Upon plotting accessibility values for genes sorted according to Rpb3 density, we find that the most and least accessible genes are in many cases not the genes with the greatest or least Rpb3 density, respectively (Fig. 5a, heatmap). Further, accessibility over the gene units and Rpb3 density over the first 500 bp exhibit a very weak Pearson correlation of 0.128. Additionally, we investigated the relationship between H2B S116C accessibility and transcription elongation, by sorting genes according to publicly available NET-Seq data, and comparing to accessibility score [49]. Similar to the results for RNAPII (rpb3), we found virtually no relationship between these parameters, with a Pearson correlation of ~ 0.039 (Additional file 1: Figure S2).

While in these analyses, we focused on nucleosome-sized DNA fragments, we considered the possibility that nucleosome loss on the most highly transcribed genes or loss of H2A/H2B dimers reduces the correlation, and that more heavily transcribed genes with one or both copies of the H2A/H2B dimer displaced due to disruption by RNAPII, would be expected to be more susceptible to enzymatic digestion resulting in DNA fragments smaller than core particle size (~ 147 bp) [48, 50]. To investigate this, we assessed possible enrichment of complexes containing DNA lengths 90 bp to 135 bp, which would correspond to digestion products from nucleosomes lacking H2A/H2B. Unlike fragment lengths corresponding to canonical nucleosomes (135 bp to 165 bp) we find that there is essentially no difference in AP/input score for subnucleosome length fragments (Additional file 1: Figure S3). Therefore, we decided to investigate the relationship between accessibility and chromatin remodelers that assist in poising genes for transcription as well as reconstituting nucleosome spacing, which mitigates cryptic transcription [51–53].

### Chromatin remodelers correlate with nucleosome surface accessibility

**Fig. 6:**
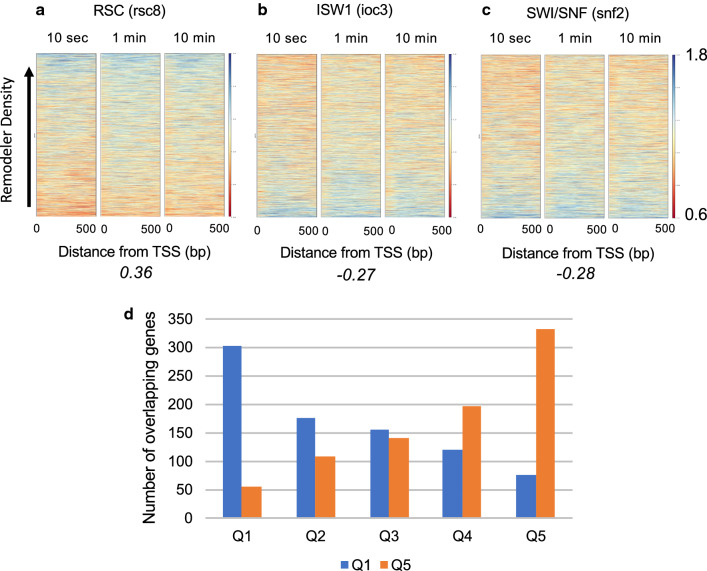
H2B S116C accessibility correlates with chromatin remodelers. Genes were sorted according to remodeler density (Rsc8, Ioc3 and Snf2) and AP/input scores were then plotted for genes preserving the remodeler sorting for RSC (**a**), ISW1a (**b**) and SWI/SNF (**c**). Average Pearson correlation coefficients for each remodeler with H2B S116C accessibility are shown below each heatmap. **d** Overlap of most accessible (Q1) and least accessible genes (Q5) from the 1 min AP/input experiment with genes sorted by RSC density. For example, ~ 300 genes which are in quintile 1 (Q1) based on accessibility, are also in Q1 for RSC density

Similar to our investigation of the relationship between accessibility and RNAPII (Rpb3 ChIP), we investigated the relationship between nucleosome accessibility and RSC, SWI/SNF and ISW1a chromatin remodelers by sorting genes according to their density of Rsc8, Snf2, and Ioc3, respectively [53], then plotted AP/input scores according to this gene order to determine whether accessibility and remodeler presence were correlated. RSC is known to restructure and evict nucleosomes within and near the promoter region to facilitate transcription [53]. Unlike Rpb3, we observe a moderate positive correlation over the first 500 bp between RSC density and accessibility scores (Pearson correlation coefficient 0.36, average of all three time points) between accessibility and Rsc8 density (Fig. 6a). In contrast, ISW1a (Ioc3), consistent with its antagonistic function with respect to RSC [53–55] exhibited an opposite, but weaker correlation with accessibility scores in the promoter proximal region (Pearson correlation coefficient of − 0.27) (Fig. 6b). Interestingly, the relationship between accessibility scores and Snf2 was similar to that of Ioc3 with an average Pearson correlation coefficient of − 0.28 (Fig. 6c). While we find that nucleosome accessibility correlates with remodeler presence, we extended the analysis described above focusing on genes showing the greatest differences in accessibility. We found quintiles 1 and 5 are much more strongly correlated to chromatin remodeler occupancy compared to quintiles 2–4, with average Pearson correlations of 0.49, − 0.37 and − 0.37 for RSC, ISW1a and SWI/SNF respectively, compared to 0.16, − 0.12 and − 0.13 for RSC, ISW1a and SWI/SNF, respectively for quintiles 2–4 (Additional file 1: Table S6). Thus, similar to the correlations between time course data sets, lower correlations for remodelers appear predominantly driven by the fact that the majority of nucleosomes and genes in yeast are equivalently accessible. In addition, chromatin remodelers are known for being difficult proteins to ChIP [56–58] and typically exhibit enrichments with less than a twofold dynamic range. We also note that RSC depletion results in movement of the − 1 and + 1 nucleosomes towards the NDR, and the change in NDR width for WT and Rsc8 depletion strains has been determined for all genes [59]. Therefore, we also investigated whether genes with NDRs affected by RSC depletion were associated with distinctive H2B S116C accessibilities. Genes were sorted according to change in NDR compared to H2B S116C accessibility data (Additional file 1: Figure S4). In contrast to the moderate positive correlation we observed with Rsc8 ChIP-seq data, we observe minimal, if any, correlation with RSC depletion-dependent change in NDR width (Additional file 1: Figure S4). These results suggest that the correlation between nucleosome surface accessibility and RSC occupancy we detected is not related to the role of RSC in defining the NDR.

### S. cerevisiae heterochromatic regions do not exhibit reduced nucleosome surface accessibility

**Fig. 7:**
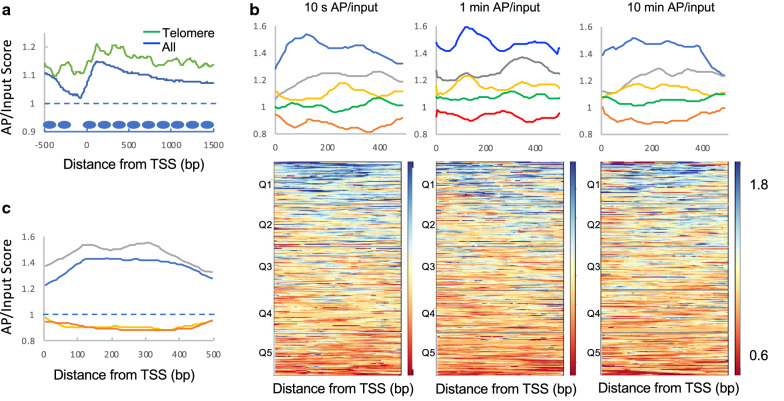
The accessibility of H2B S116C in heterochromatic regions is commensurate with the genomic average. AP/input scores were determined for genes located within 20 kb of telomeres. **a** Comparison of aggregate scores for telomere-proximal genes (green line) and all genes (blue line). Nucleosomes positions are shown as in Fig. 1a. **b** Quintile analysis for three time points of BM modification. Quintiles 1–5 are plotted with blue, gray, yellow, green and red lines, respectively. Heat maps show individual quintiles. **c** Comparison of average scores for quintile 1 (telomere, gray; all genes, blue) and quintile 5 (telomere, orange; all genes, red)

It is well known that the *S. cerevisiae* genome is largely euchromatic, however there are heterochromatic regions where gene expression is repressed. Genes within 20 kb of the telomeres have been found to be less transcriptionally active and in some instances are bound by the SIR complex which induces heterochromatin formation in *S. cerevisiae* [60–62]. Although found to produce far fewer transcripts compared to non-subtelomeric genes, we find that these genes exhibit overall accessibility scores that are slightly greater than those for all genes (Fig. 7a). Moreover, the ranges of scores were comparable to that of average genes, with ranked quintiles exhibit comparable accessibility profiles compared to all genes (Fig. 7b). Indeed, a comparison of accessibility scores for genes within the top and bottom quintiles for telomeres exhibit identical overall range compared to those for all genes (Fig. 7c). Moreover, similar to all genes, accessibility of telomere-proximal genes was uncorrelated with Rpb3 (Pol II) density (Pearson coefficient − 0.02). While initially surprising, this finding is in line with previous studies suggesting that *S. cerevisiae* heterochromatin does not silence gene expression via steric occlusion [63–65]. It should also be noted that the different modification times did not impact these findings. Our results for the H2B S116C surface site suggest that the chromatin in *S. cerevisiae* is not binary (i.e., open vs. condensed), but rather that changes in accessibility are more subtle, possibly as a consequence of nucleosome remodeling and positioning. To ensure that our standard nuclei resuspension conditions (buffer containing 75 mM NaCl and 0.5 MgCl_2_) maintained folded structures, we repeated this experiment for the 1 min modification time point, using a nuclear resuspension buffer containing spermidine (20 mM PIPES pH 6.3, 1 M sorbitol, 0.2 mM spermidine) as polyamines have been shown to stabilize higher-order chromatin structures [66–69]. We find that these conditions yield similar results for remodeler density, transcriptional activity as gauged by Rpb3 density and for subtelomere gene accessibilities (Additional file 1: Figure S5A–C).

### Internal nucleosome surface sites are not significantly exposed genome-wide in yeast nuclei

Previous reports indicated that nucleosomes isolated from transcriptionally active chromatin in *S. cerevisiae* at least transiently expose histone surfaces normally buried within the canonical nucleosome. For example, when present, the thiol group of H3 C110, which is normally buried within the 4-helix bundle comprising the H3–H3 interface at the center of the nucleosome, was found to be accessible to organomercural-agarose columns in nucleosomes derived from transcriptionally active genes in yeast [70, 71]. In addition, H2A/H2B dimers are known to exhibit rapid turnover in transcriptionally active regions, suggesting the possibility that H2A/H2B-(H3/H4)_2_ hexamers and (H3/H4)_2_ tetramers may transiently exist in genes being transcribed by RNAPII [50, 72, 73]. We therefore generated yeast cells in which H3 was replaced with either H3 S102C or H3 A110C, which are buried within the canonical nucleosome structure to determine whether the H2A–H2B–hexamer interface or H3–H3 interface, respectively, are exposed genome-wide in a manner that correlates with gene expression. Because such altered nucleosomes are likely to comprise a minor proportion of nucleosomes at any point in time, we opted for a modification of 10 min to maximize detection. As nucleosomes with exposed internal surfaces are likely to be more rapidly digested to sub-nucleosomal products compared to bulk nucleosomes, we first examined enrichments from nucleosomes prepared under reduced MNase digestions conditions in which some dinucleosome and trinuclesome length DNA fragments remains, compared to primarily mononucleosome-sized fragments generated under standard conditions (Additional file 1: Figure S6A). Following library preparation of input and AP samples, we observed that there was no detectable enrichment of nucleosomes in the AP fraction from either mutant with respect to wild type (Table 1), with yeast/mouse DNA ratios for the mutants less than that of wild type (Additional file 1: Table S7, top). However, in this experiment where less extensive MNase digestion conditions were employed, we observed yeast/mouse ratios slightly lower than expected, suggesting the less digested chromatin yielded increased background by preserving non-histone chromatin binding protein associations with the DNA. Therefore, we repeated this experiment using chromatin that was subjected to more extensive MNase digestion yielding predominantly ‘core particle’ length DNA fragments (Additional file 1: Figure S6B). While the more extensive digestion resulted in yeast/mouse control ratios in line with expectation (Additional file 1: Table S7, bottom), similar to the under-digested chromatin, we found that yeast/mouse DNA rations of both mutants were slightly less than that of wild type suggesting that the recovered yeast DNA was background. We considered the possibility that exposure of these internal sites would involve disruption of the canonical nucleosome structure and thus more extensive trimming of DNA within such complexes by MNase. However, we were unable to detect enrichment for shorter DNA lengths attributable to tetramer or hexamer histone-DNA complexes (Additional file 1: Figure S6A, S6B). Thus, for all digestion conditions attempted, we find that both of these internal mutants return DNA commensurate with background (Table 1 and Additional file 1: Table S7). Given that we were able to routinely achieve 25- to 50-fold enrichment for the H2B S116C surface site (see above), with < 5% of the nucleosomes modified, these data suggest that < 0.25% of all nucleosomes, we were unable to achieve enrichment above background with H3 S102C or H3 A110C.

**Table 1:** Fold enrichment of mutant H3 internal nucleosome sites

Sample	MNase	AP/input	Fold enrichment
WT H3	Low	0.068	1.0
H3 S102C	Low	0.044	0.65
H3 A110C	Low	0.042	0.62
WT H3	Medium	0.018	1.0
H3 S102C	Medium	0.017	0.94
WT H3	High	0.093	1.0
H3 S102C	High	0.045	0.49
H3 A110C	High	0.054	0.58

Fold enrichment is calculated by dividing the ratio of AP/input of the mutant by that of the wild type. The AP/input ratios are determined by calculating the ratio of yeast:mouse aligned reads for each sample. Fold-enrichment values of ~ 1 or less indicate no enrichment for the internal H3 mutants

## Discussion

We employ a new method [36] to probe the accessibility of external and internal protein surfaces in nucleosomes genome-wide. We find that a cysteine installed on the nucleosome protein surface is widely exposed in the genome of *S. cerevisiae*. We anticipated that this site would be more exposed in open, transcribed euchromatic regions of the genome, but also exhibit reduced exposure, especially within known regions of heterochromatin and inactive genes. However, we find similar average accessibilities for nucleosomes within 0.5 kb upstream to 1.5 kb downstream with respect to the TSS of all yeast genes, including those within sub-temomeric heterochromatin regions. Somewhat greater variability in accessibility was found in ‘promoter proximal’ nucleosomes located within the first 500 bp of genes, but even in this case the vast majority of genes exhibited accessibilities that differed by less than twofold. We designed our conditions to maintain the integrity of yeast chromatin in isolated nuclei, consistent with in vitro work demonstrating maximally folded chromatin states at salt concentrations commensurate with those used in this study [7]. Moreover, to confirm maintenance of fully compacted yeast chromatin we repeated the experiment with a spermidine buffer shown to achieve maximal compaction of chromatin in yeast nuclei [74], and found results identical to those obtained with our standard conditions. Thus, our data indicate that the external nucleosome surface accessibility as interrogated via H2B S116C is widely accessible throughout *S. cerevisiae* nuclei. Our findings are consistent with studies indicating minimal long-range inter-nucleosomal interactions in *S. cerevisiae* and that regulation of *S. cerevisiae* chromatin is regulated largely by local interactions [13, 75, 76].

Our data also indicate that diffusion of BM through the nucleus occurs much more rapidly than cysteine modification, because accessibility differences are not affected by extent of modification. We find a high degree of correlation between AP data sets from 10 s and 10 min of modification (Pearson correlation 0.86), despite the large difference in the extent of modification between these two experiments (Fig. 1b). Moreover, all genes have nearly equivalent average scores based on their AP 10 s/AP 10 min ratio, indicating no subset of genes is systematically distinct (results not shown). These results indicate that nucleosomes exhibit a characteristic relative extent of modification regardless of the overall level of reaction, and that rate of BM diffusion does affect these values.

The vast majority of the budding yeast genome is genic, euchromatic and heavily acetylated and therefore would not be expected to possess long stretches of highly condensed chromatin. Our investigation of heterochromatic regions, which would presumably be most likely to contain higher-order chromatin structures involving ‘face-to-face’ nucleosome packing indicates that H2B S116C remains readily accessible. Analysis of genes within 20 kb of the telomeres yield accessibility scores that are on par with that observed for the remainder of the *S. cerevisiae* genome. Recent work has shown that *S. cerevisiae* heterochromatin, facilitated by Sir proteins, does condense but not to the extent of an oligonucleosome array in magnesium [77]. Additionally, it has been shown that *S. cerevisiae* heterochromatin does not hinder transcription through steric occlusion but rather specifically targets and prevents the transcriptional machinery from associating with the DNA [63, 64]. Early investigation into yeast chromatin structure found that the transcriptionally active DNA was as sensitive to DNase I digestion as the rest of the genome [78]. More recently, it was found that accessibility of DNA for euchromatic and heterochromatic regions were equivalent, commensurate with our finding that nucleosome external surface accessibilities were similar between these two chromatin states [65]. Although an earlier study identified higher-order chromatin structure in *S. cerevisiae*, more recent studies investigating *S. cerevisiae* chromatin structure did not identify long-range inter-nucleosomal interactions, indicative of higher-order chromatin structures, using electron cryotomography or chromatin conformation techniques [13, 75, 76, 79–81]. Additionally, it has been shown that organisms with greater nucleosome repeat lengths (~ 197 bp or greater) more readily form compact chromatin structures including the 30 nm fiber as compared to shorter nucleosome repeat lengths (167 bp) [82]. Therefore, it is not surprising that us and others do not observe higher order chromatin structures in *S. cerevisiae* given that the nucleosome repeat length for this organism is ~ 165 bp [83].

We find that the accessibilities of nucleosomes within the first 500 bp of gene transcription units exhibit a greater dynamic range, with approximately 50% difference between the least and most accessible quintiles. While H2B S116C accessibility appears to be poorly correlated with Pol II levels, accessibility is moderately correlated with the presence of the RSC chromatin remodeler, exhibiting weaker negative correlations with SWI/SNF and ISW1a. In addition, we found no correlation between nucleosome surface accessibility and the effect of RSC on NDR width, suggesting other RSC functions are relevant to accessibility. It is interesting to note that while RSC depletion results in a loss of transcriptional activity as shown by total RNA analysis and a distinct change in transcription profile, RSC depletion does not alter overall nucleosome spacing or drastically change Pol II occupancy patterns [54, 84, 85]. Moreover, we find that transcription detected by NET-Seq shows little correlation with accessibility scores. These data are consistent with results indicating that RSC is weakly enriched on active genes [52]. Based these results we propose that RSC poises genes for transcription by increasing nucleosome accessibility prior to Pol II binding. After Pol II binding and initiation, the process of transcription results in nucleosome disruption along with rapid nucleosome reformation to limit cryptic transcription [86–90], resulting in less accessible chromatin on actively transcribed genes compared to genes poised for transcription. This effect may be due to nucleosome spacing, as most active genes have fewer nucleosomes and increased sub-nucleosomes [48], while the remaining nucleosomes have very short spacing which may reduce their accessibility [108]. Furthermore, nucleosomes and subnucleosomes located within the vicinity of promoters have been shown to associate with RSC, which may facilitate local exposure of the nucleosome surface [91, 92]. Indeed, analysis of restriction enzyme access and digestion of chromatin in *S. cerevisiae* and mouse hepatocytes showed that DNA accessibility is predominantly influenced by nucleosome spacing, and that enzyme accessibility is not correlated with gene activity [65].

Given the similar role to RSC in promoting gene expression, it was somewhat surprising to observe a negative correlation between SWI/SNF and nucleosome surface accessibility, similar to that observed between accessibility and ISWa. We note that RSC and SWI/SNF exhibit a negative correlation among themselves (− 0.337), and that ISW1 and SWI/SNF occupancies are well correlated (0.682). Because SWI/SNF is much less abundant than RSC and associated with a distinct cohort of genes compared to RSC, it is possible that the negative correlation is due to a lack of correspondence between the most accessible genes and those with greatest SWI/SNF density. SWI/SNF is an important component for transcriptional activation [93–97]. Therefore, it is possible that these genes are in a preliminary state of activation, being primed by SWI/SNF remodeling with downstream nucleosomes still maintaining regular spacing, limiting their accessibility. This is in contrast to RSC which has been shown to be a factor in transcription elongation [54, 98, 99]. Thus, it is possible that the accessibility of the external nucleosome surface in *S. cerevisiae* is dependent upon the transcriptional state (initiation vs. elongation) and more directly, the level of chromatin remodeling during these two states.

In stark contrast to the H2B S116C external surface site, we find little evidence of accessibility of two internal nucleosome sites installed on H3. Two residues on H3, H3 102 and H3 110, were selected to investigate the accessibility of internal nucleosome surfaces. Interestingly, prior studies have suggested that the H3–H3 interface in nucleosomes from transcriptionally active regions of chromatin is highly accessible in yeast, as well as chicken and HeLa cells [70, 71, 100, 101]. These studies selected for reactivity of H3 A110C (or H3 containing native cysteine at the 110 position) in preparations of nucleosome core particles (NCPs) using organomercurial affinity columns finding ~ 20% of the total population of yeast nucleosomes were retained due to reaction with the thiol group within H3 A110C. Notably, it was reported that selected population contained nearly stoichiometric ratios of H2A/H2B dimers and H3/H4 tetramers and DNA fragment lengths typical for NCPs, suggesting little or no overall deviation from canonical nucleosome structure, as might be expected for nucleosomes with exposed H3-H3 interfaces [71]. In contrast, our experiments probed H3 C110 accessibilities in yeast nuclei containing unperturbed chromatin, and found no evidence for widespread non-canonical exposure of the H3–H3 interface. From experiments with H2B S116C mutants, we estimate that modification of ~ 4% of nucleosomes in yeast nuclei resulted in an average of ~ 40-fold enrichment (Table 1, 1 min and 10 min modifications), while modification for 10 s resulted in about 0.4% of nucleosomes modified and yielded much smaller enrichments, about fivefold, but with signal still well above background. Assuming we could detect enrichment of half this amount of modification of H3 A110C nucleosomes, we estimate that no more than 0.2% of nucleosomes exist with exposed H3-H3 interfaces in native yeast nuclei clearly much less than the ~ 20% detected in previous studies [70]. This discrepancy may be due to weakening of the H3/H3 interface in the prior preparations of NCPs due to exposure to the organomecurial resin or other effects of the preparation of the NCPs from yeast nuclei, whereas we probed nucleosomes in native chromatin. Nevertheless, our data imply that the vast majority of nucleosomes within actively transcribed regions in yeast nuclei do not exhibit stable exposure of the H3-H3 interface, but rather exist as canonical nucleosomes.

We also probed for H3 102, which is located at the H2A–H2B/H3–H4 interface and is exposed following removal of H2A–H2B dimer. RNA polymerase II transcription occurs with transient disruption of the canonical nucleosome structure, resulting in H2A–H2B dimer loss and, in the case of very high densities of RNAPII, even octamer eviction [48, 50, 72, 73, 102–105]. Additionally, heavily transcribed genes are more prone to lose nucleosome phasing, with reduced spacing and nucleosome occupancy [106–110]. While transcription does disrupt canonical nucleosome structure, nucleosomes are rapidly reformed, primarily resulting in exchange of H2A/H2B dimers with free pools, along with some exchange of H3/H4 tetramers [73, 105, 111–114]. Our results are consistent with the idea that chromatin reformation in the wake of transcribing RNAPII is extremely efficient.

In addition, such noncanonical nucleosome structures are expected to survive nuclei preparation and subsequent steps including washing of MNase digested chromatin fragments bound to the streptavidin-agarose resin, as H3–H4 tetramer dissociation from DNA occurs at ~ 1.2 M NaCl [115–120]. Therefore, our wash buffers containing 0.15 and 0.6 M NaCl should not promote significant dissociation of DNA from resin-bound H3–H4 tetramers containing modified H3 S102C or H3 A110C.

## Conclusions

Overall, we find that an external nucleosome surface site, H2B S116C, is widely accessible throughout the *S. cerevisiae* genome, including sub-telomeric regions, suggesting limited inter-nucleosomal interactions mediated by these surfaces. Furthermore, we find that the accessibility is modestly correlated with chromatin remodelers: RSC, ISW1a and SWI/SNF. RSC and accessibility exhibit a positive correlation whereas ISW1a and SWI/SNF exhibits a negative correlation. These results may indicate that for yeast, proper nucleosome spacing may be a primary driver of yeast chromatin structure and gene regulation. Consistent with this idea, we find that the accessibility of nucleosomes closest to the promoter is more dynamic than nucleosomes found upstream of the promoter and further downstream within gene bodies. Conversely, we find no evidence of widespread exposure of internal nucleosome surfaces suggesting that disruption of the canonical nucleosome structure and subsequent exposure of these sites is an extremely transient process.

## Supplementary Information

**Additional file 1: Figure S1.** Differences in accessibility scores (AP/input) seem to be influenced more by differences in AP reads (right) rather than input (left). Input and AP reads were plotted for each quintile. The range of AP scores is greater than that of the inputs which do not differ much between quintiles. **Figure S2.** Weak correlation between H2B S116C accessibility and nascent transcript (NET-seq) density. Genes were sorted according to NET-seq score and grouped into quintiles where quintile 1 contained the genes with the greatest nascent transcript density and quintile 5 the least. AP/input scores were then plotted for genes preserving the NET-seq sorting and grouping. All time points exhibit similar ordering and profiles and the difference between quintiles is minor. **Figure S3.** H2B S116C accessibility and RNAPII density are not correlated for subnucleosome length fragments (90–135 bp). Genes were sorted according to rpb3 density and grouped into quintiles where quintile 1 contained the genes with the greatest rpb3 density and quintile 5 the least as in Fig. 2. Only DNA fragments which were 90–135 bp were included to investigate noncanonical nucleosomes which may be absent H2A–H2B dimer. **Figure S4.** Nucleosome external surface accessibility does not exhibit obvious correlation with genes most affected by Rsc8 depletion. Genes were sorted according to change in NDR width following Rsc8 depletion (ref. [59]). This gene order was preserved and accessibility values for each time point was plotted from − 500 to 500 bp with respect to TSS. In contrast to the ChIP-seq data (Fig. 6), the positive correlation is no longer observed returning Spearman correlation coefficients of 0.06, 0.04 and 0.04 for 10 s, 1 min and 10 min, respectively. Note that spearman correlation was used here and is for TSS to + 500 bp as the NDR width data were provided as rank of NDR width change. We find that Pearson and Spearman correlation coefficients are highly similar with our data. **Figure S5.** Alternative nuclei resuspension buffer yields all similar results for H2B S116C accessibility. A. Nuclei were resuspended in alternative buffer (20 mM PIPES pH 6.3, 1 M sorbitol, 0.2 mM spermidine) to ensure folded chromatin structures were maintained. Accessibility scores were plotted according to remodeler density as in Fig. 6 and display similar correlations (Pearson correlations in italics above respective heatmap). B. Genes were sorted according to rpb3 density and grouped into quintiles as in Fig. 5. C. Average H2B S116C accessibility scores for all PolII genes and subtelomere genes as in Fig. 7. **Figure S6.** Extent of desired MNase digestion for internal H3 mutants. To preserve noncanonical nucleosome structures, accessibility of H3 mutants were investigated using less digested chromatin where the predominant species was greater than core particle size (~ 147 bp). In S6A, digestion yielded predominantly mononucleosomes with ~ 20 bp of total linker DNA with the major peak being ~ 170 bp. In S6B, digestion conditions were used generating core particle length species with the major peak at ~ 150 bp, similar to digestions for the H2B surface mutant. **Table S1.** Sequencing summary statistics for H2B S116C biotin-maleimide time course experiment. The total number of aligned reads for all samples is on the same order of magnitude [106]. However, the vast majority of the aligned reads for wild-type AP samples are found to be from the mouse DNA spike in control. Consequently, the ratio of aligned yeast reads to aligned mouse reads notably greater for the H2B S116C AP samples compared to the wild-type AP samples. **Table S2.** Fold enrichment and fraction of modified nucleosomes from WT and H2B S116C yeast nuclei. Fold enrichment was calculated by dividing AP/input of the mutant by that of the wild type. The AP/input ratio is determined by calculating the ratio of yeast:mouse aligned reads for each sample. The percentage of modified nucleosomes was determined by dividing the amount of AP DNA by the corrected input as determined in Tables S1, S2. All time points reveal signal above background with 1 min and 10 min time points averaging a fold enrichment of 43. Percent of modified nucleosomes is corrected by subtracting the average apparent enrichment in the WT samples (0.08). Rep indicates replicate experiments. Bolded numbers are plotted in Fig. 1a. **Table S3.** Sequencing summary statistics for H2B S116C biotin-maleimide time course replicate experiments. The total number of aligned reads for all samples is on the same order of magnitude [106]. However, the vast majority of the aligned reads for wild-type AP samples are found to be from the mouse DNA spike in control. Consequently, the ratio of aligned yeast reads to aligned mouse reads notably greater for the H2B S116C AP samples compared to the wild-type AP samples. **Table S4.** Promoter-proximal nucleosomes exhibit greater differences in accessibility as compared to nucleosomes across genes. AP/input accessibility scores were calculated for genes in two different windows, − 500 to + 1500 (A) and TSS + 500 (B). Accessibility scores are more dynamic for promoter-proximal nucleosomes as compared to the nucleosomes throughout the region − 0.5 kb to + 1.5 kb with respect to the TSS. **Table S5.** Pearson correlation coefficients for accessibility scores within 500 bp of TSS. The average AP/input scores for each gene were determined from TSS to 500 bp and the Pearson correlation was determined between each data set. Overall, these values are similar to those found from TSS to TTS (Fig. 1b). **Table S6.** Nucleosome accessibility correlate with remodeler density. Pearson correlations were determined for accessibility scores and remodeler density for all genes (A). Additionally, genes were grouped into quintiles based on remodeler density. Pearson correlations were then determined for accessibility and remodeler density scores for quintiles 1 and 5 (B) and quintiles 2–4 (C). **Table S7.** Sequencing summary statistics for internal nucleosome surface mutant sites. Yeast nuclei were modified and input and AP-selected nucleosome libraries prepared and sequenced as described in the text. 1 ng of mouse DNA was added as a normalization control to all samples. Note the yeast/mouse DNA ratio in wild-type samples exceeds that of mutants suggesting no enrichment above background.

## Data Availability

The datasets used and/or analyzed during this current study are available from the corresponding author on reasonable request.
