# Supplementary material for: Global histone protein surface accessibility in yeast indicates a uniformly loosely packed genome with canonical nucleosomes
Source: Epigenetics Chromatin. 2021 Jan 11;14:5. doi: 10.1186/s13072-020-00381-5 (PMC7802155; doi:10.1186/s13072-020-00381-5)
Supplement: Supplementary file 1 — Additional file 1: Figure S1. Differences in accessibility scores (AP/input) seem to be influenced more by differences in AP reads (right) rather than input (left). Input and AP reads were plotted for each quintile. The range of AP scores is greater than that of the inputs which do not differ much between quintiles. Figure S2. Weak correlation between H2B S116C accessibility and nascent transcript (NET-seq) density. Genes were sorted according to NET-seq score and grouped into quintiles where quintile 1 contained the genes with the greatest nascent transcript density and quintile 5 the least. AP/input scores were then plotted for genes preserving the NET-seq sorting and grouping. All time points exhibit similar ordering and profiles and the difference between quintiles is minor. Figure S3. H2B S116C accessibility and RNAPII density are not correlated for subnucleosome length fragments (90–135 bp). Genes were sorted according to rpb3 density and grouped into quintiles where quintile 1 contained the genes with the greatest rpb3 density and quintile 5 the least as in Fig. 2. Only DNA fragments which were 90–135 bp were included to investigate noncanonical nucleosomes which may be absent H2A–H2B dimer. Figure S4. Nucleosome external surface accessibility does not exhibit obvious correlation with genes most affected by Rsc8 depletion. Genes were sorted according to change in NDR width following Rsc8 depletion (ref. [59]). This gene order was preserved and accessibility values for each time point was plotted from − 500 to 500 bp with respect to TSS. In contrast to the ChIP-seq data (Fig. 6), the positive correlation is no longer observed returning Spearman correlation coefficients of 0.06, 0.04 and 0.04 for 10 s, 1 min and 10 min, respectively. Note that spearman correlation was used here and is for TSS to + 500 bp as the NDR width data were provided as rank of NDR width change. We find that Pearson and Spearman correlation coefficients are highly similar with our data. F [file 13072_2020_381_MOESM1_ESM.pptx]

## Slide 1
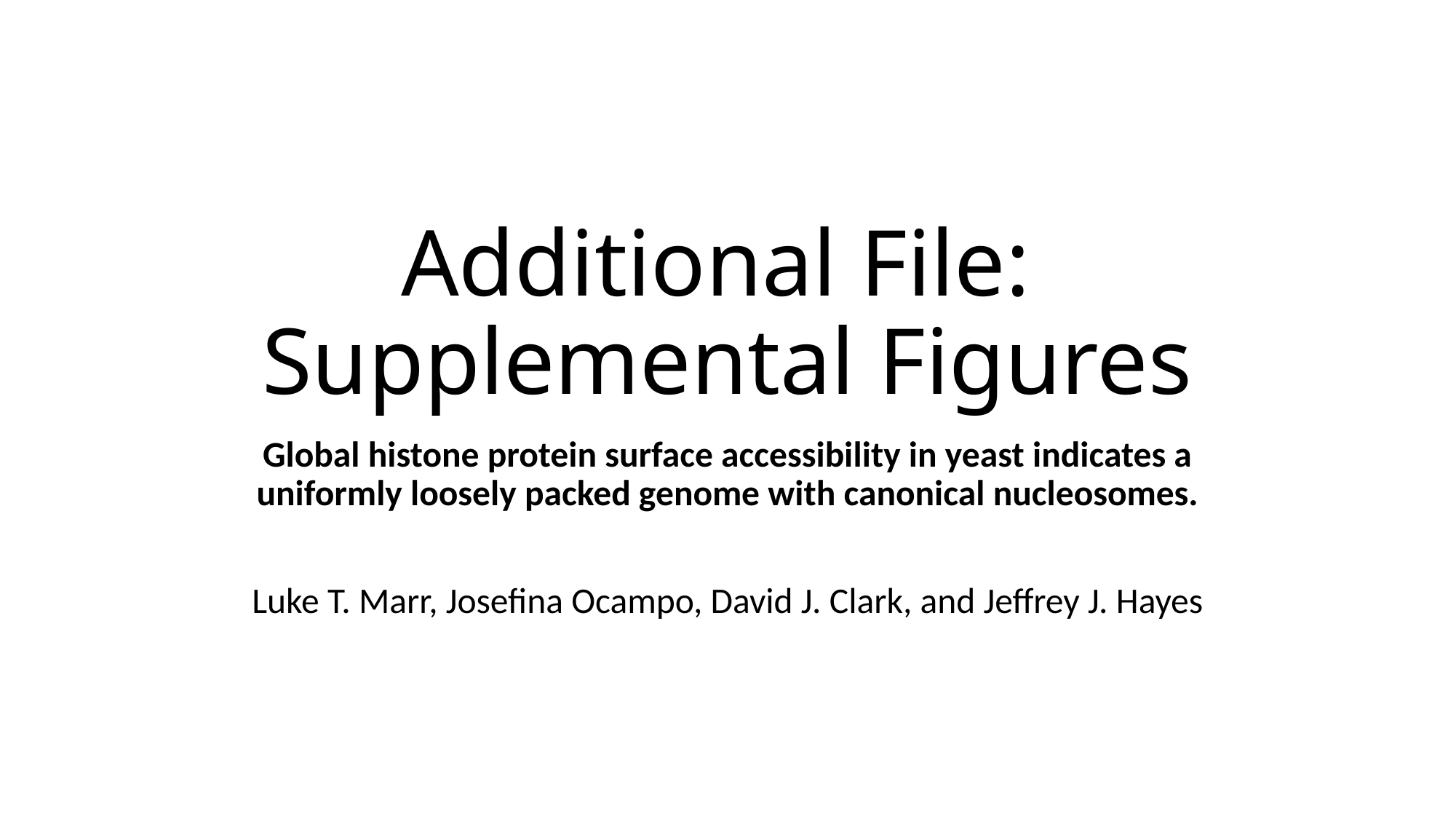

# Additional File: Supplemental Figures
Global histone protein surface accessibility in yeast indicates a uniformly loosely packed genome with canonical nucleosomes.
Luke T. Marr, Josefina Ocampo, David J. Clark, and Jeffrey J. Hayes

## Slide 2
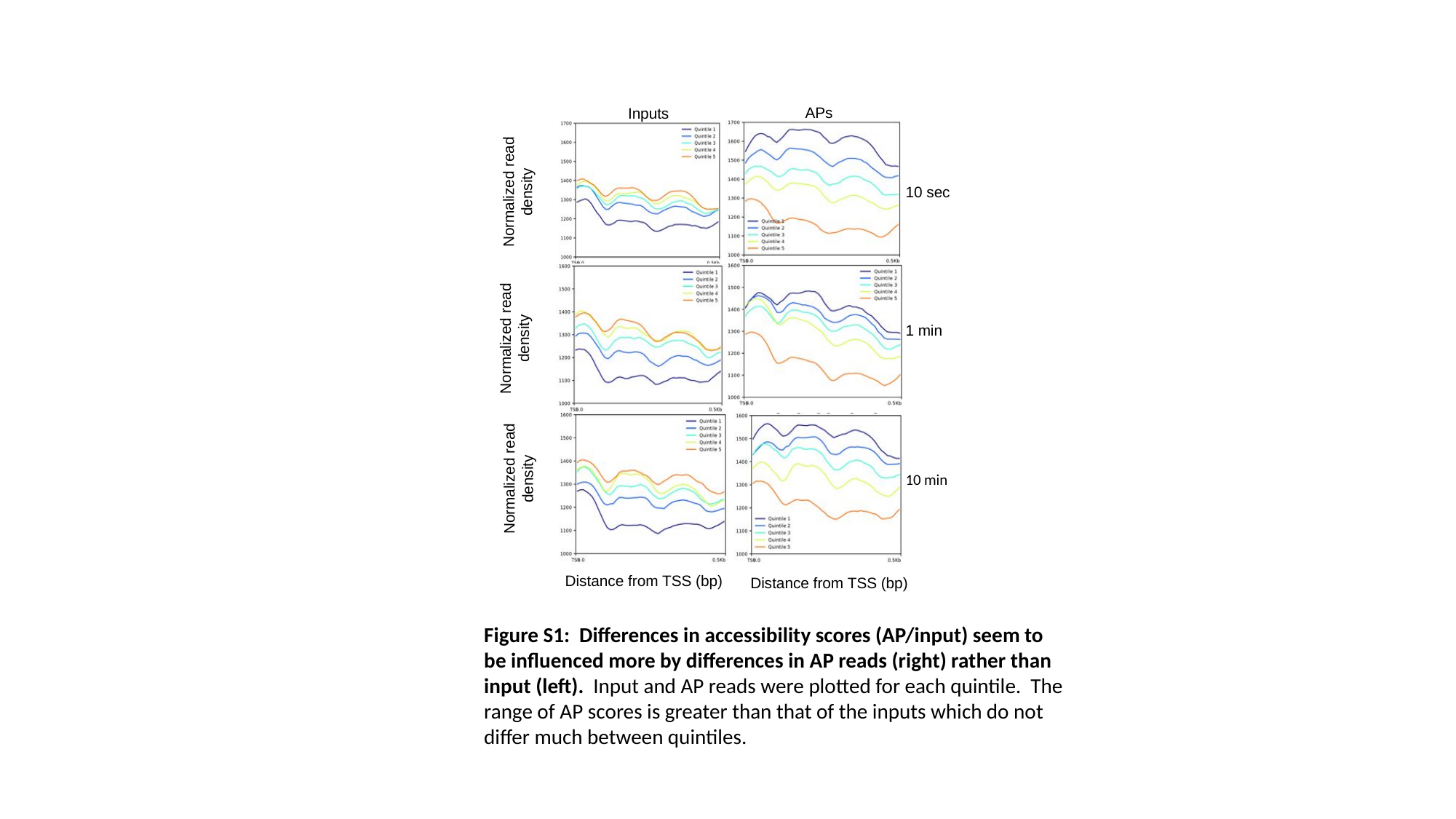

APs
Inputs
10 sec
10 min
1 min
Normalized read density
Normalized read density
Normalized read density
Distance from TSS (bp)
Distance from TSS (bp)
Figure S1: Differences in accessibility scores (AP/input) seem to be influenced more by differences in AP reads (right) rather than input (left). Input and AP reads were plotted for each quintile. The range of AP scores is greater than that of the inputs which do not differ much between quintiles.

## Slide 3
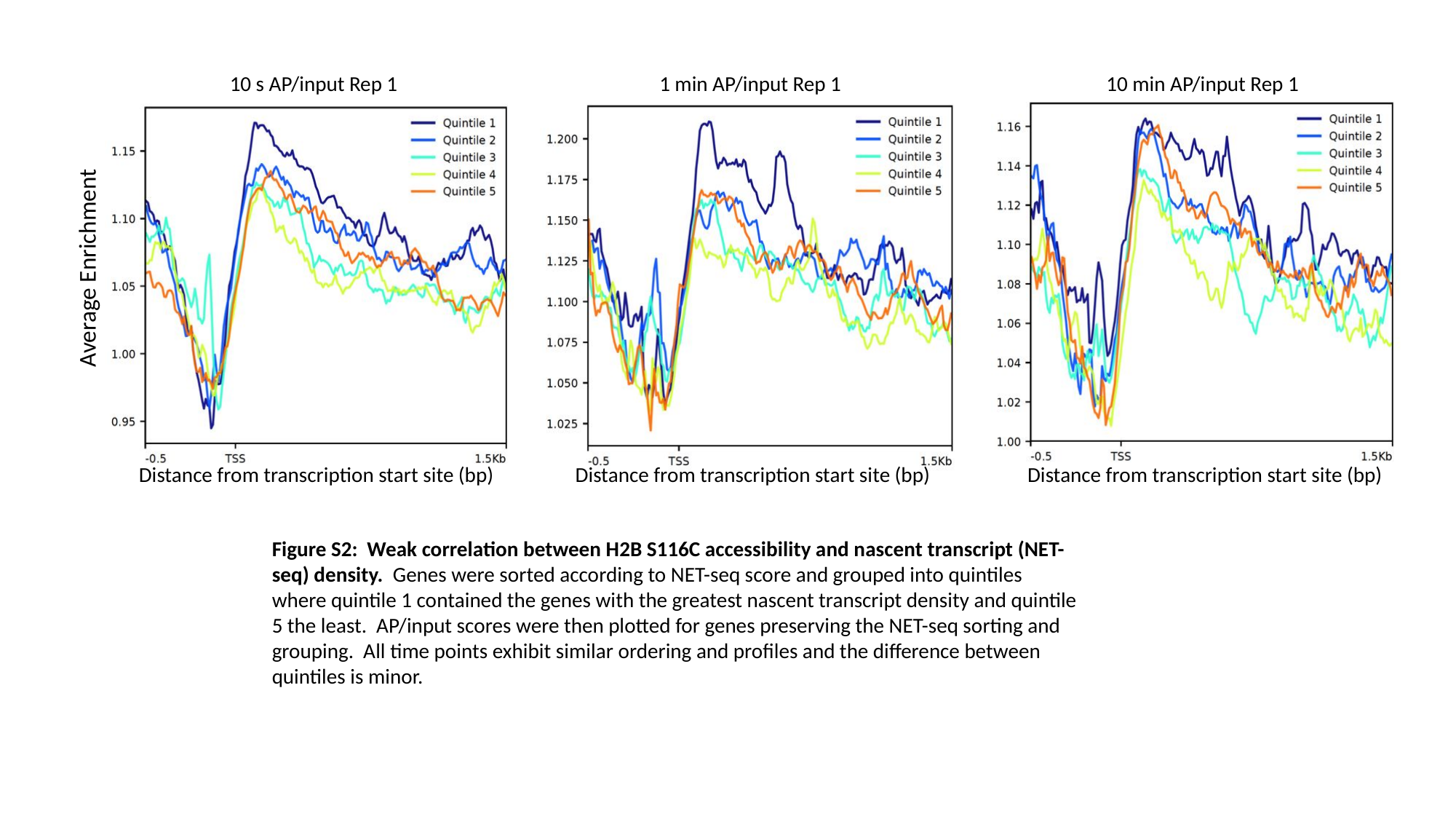

10 s AP/input Rep 1
1 min AP/input Rep 1
10 min AP/input Rep 1
Average Enrichment
Distance from transcription start site (bp)
Distance from transcription start site (bp)
Distance from transcription start site (bp)
Figure S2: Weak correlation between H2B S116C accessibility and nascent transcript (NET-seq) density. Genes were sorted according to NET-seq score and grouped into quintiles where quintile 1 contained the genes with the greatest nascent transcript density and quintile 5 the least. AP/input scores were then plotted for genes preserving the NET-seq sorting and grouping. All time points exhibit similar ordering and profiles and the difference between quintiles is minor.

## Slide 4
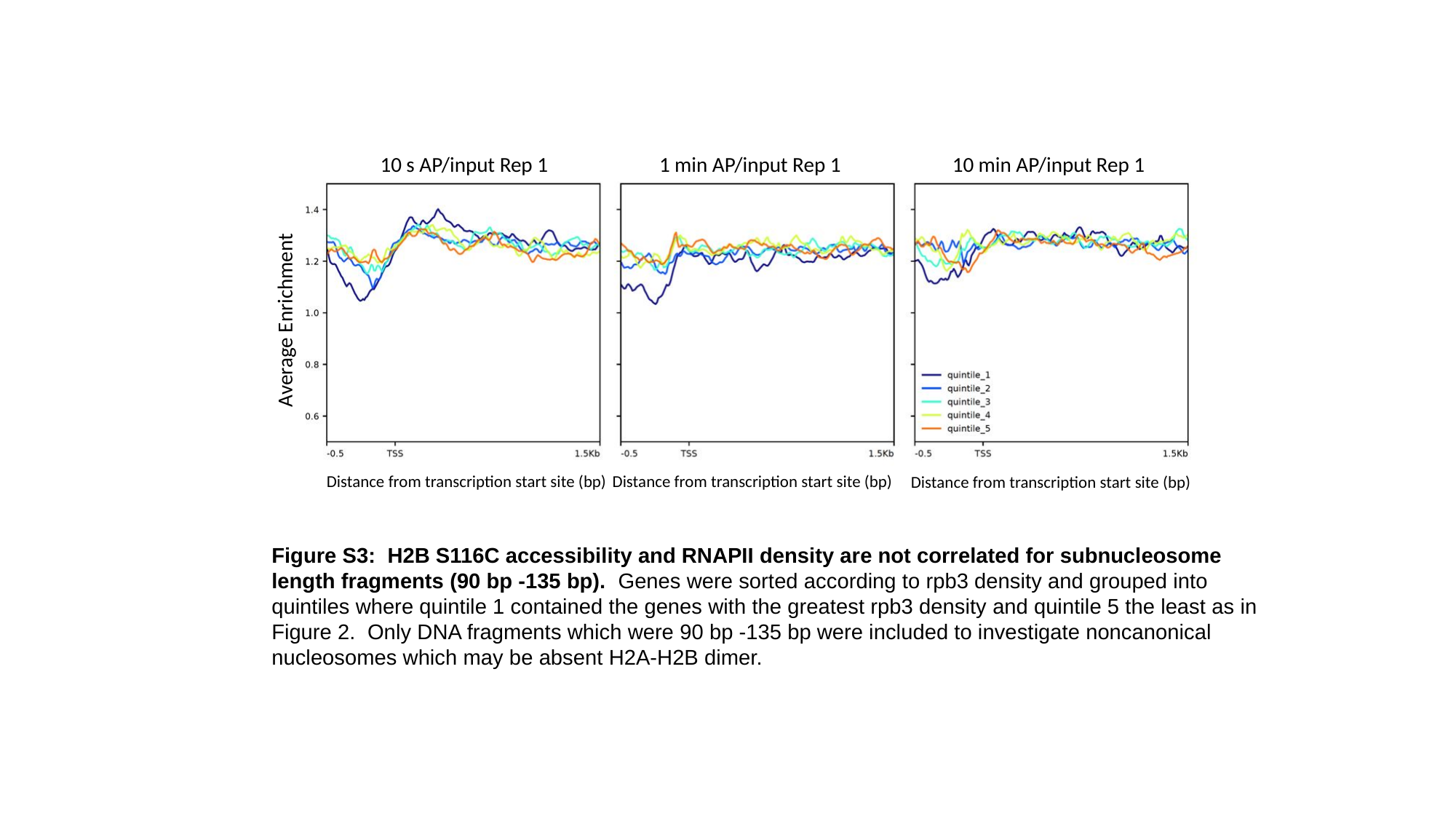

10 s AP/input Rep 1
10 min AP/input Rep 1
1 min AP/input Rep 1
Average Enrichment
Distance from transcription start site (bp)
Distance from transcription start site (bp)
Distance from transcription start site (bp)
Figure S3: H2B S116C accessibility and RNAPII density are not correlated for subnucleosome length fragments (90 bp -135 bp). Genes were sorted according to rpb3 density and grouped into quintiles where quintile 1 contained the genes with the greatest rpb3 density and quintile 5 the least as in Figure 2. Only DNA fragments which were 90 bp -135 bp were included to investigate noncanonical nucleosomes which may be absent H2A-H2B dimer.

## Slide 5
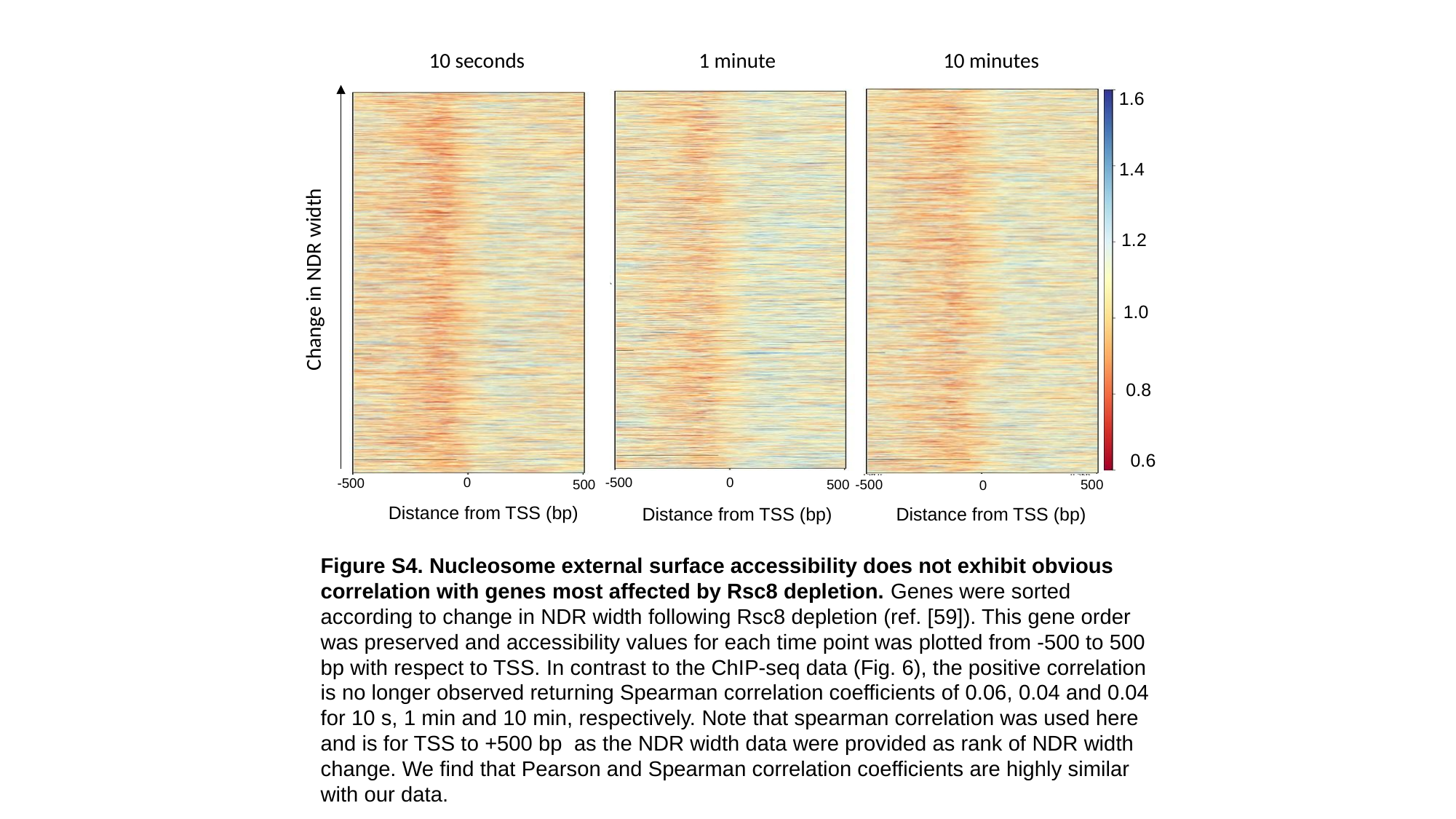

1 minute
10 minutes
10 seconds
1.6
1.4
1.2
1.0
0.8
0.6
-500
-500
-500
500
500
500
Distance from TSS (bp)
Distance from TSS (bp)
Distance from TSS (bp)
Change in NDR width
0
0
0
Figure S4. Nucleosome external surface accessibility does not exhibit obvious correlation with genes most affected by Rsc8 depletion. Genes were sorted according to change in NDR width following Rsc8 depletion (ref. [59]). This gene order was preserved and accessibility values for each time point was plotted from -500 to 500 bp with respect to TSS. In contrast to the ChIP-seq data (Fig. 6), the positive correlation is no longer observed returning Spearman correlation coefficients of 0.06, 0.04 and 0.04 for 10 s, 1 min and 10 min, respectively. Note that spearman correlation was used here and is for TSS to +500 bp as the NDR width data were provided as rank of NDR width change. We find that Pearson and Spearman correlation coefficients are highly similar with our data.

## Slide 6
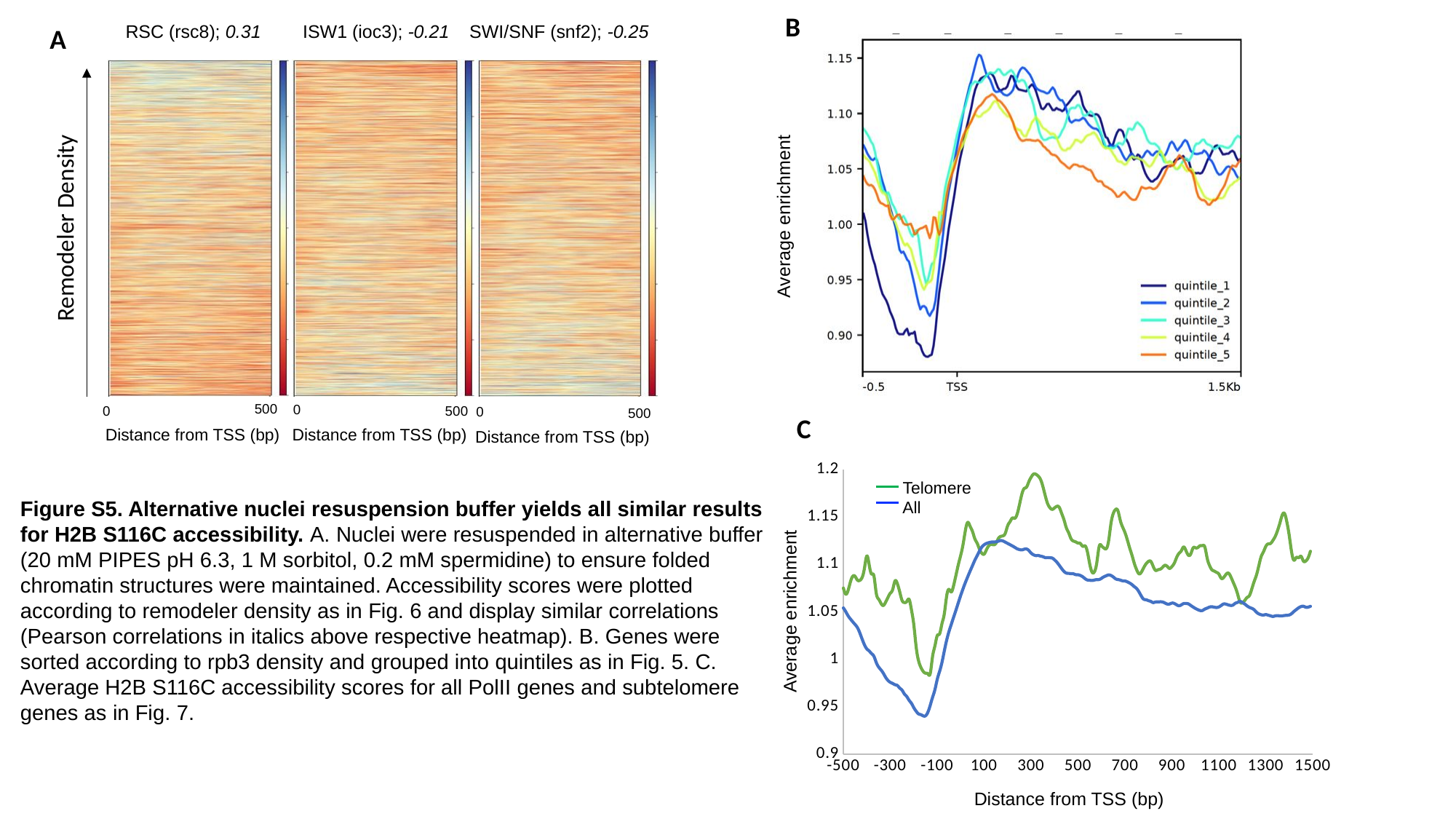

B
RSC (rsc8); 0.31
ISW1 (ioc3); -0.21
SWI/SNF (snf2); -0.25
Remodeler Density
500
0
Distance from TSS (bp)
500
0
Distance from TSS (bp)
0
500
Distance from TSS (bp)
A
Average enrichment
C
### Chart
| Category | | |
|---|---|---|Telomere
All
Figure S5. Alternative nuclei resuspension buffer yields all similar results for H2B S116C accessibility. A. Nuclei were resuspended in alternative buffer (20 mM PIPES pH 6.3, 1 M sorbitol, 0.2 mM spermidine) to ensure folded chromatin structures were maintained. Accessibility scores were plotted according to remodeler density as in Fig. 6 and display similar correlations (Pearson correlations in italics above respective heatmap). B. Genes were sorted according to rpb3 density and grouped into quintiles as in Fig. 5. C. Average H2B S116C accessibility scores for all PolII genes and subtelomere genes as in Fig. 7.
Average enrichment
Distance from TSS (bp)

## Slide 7
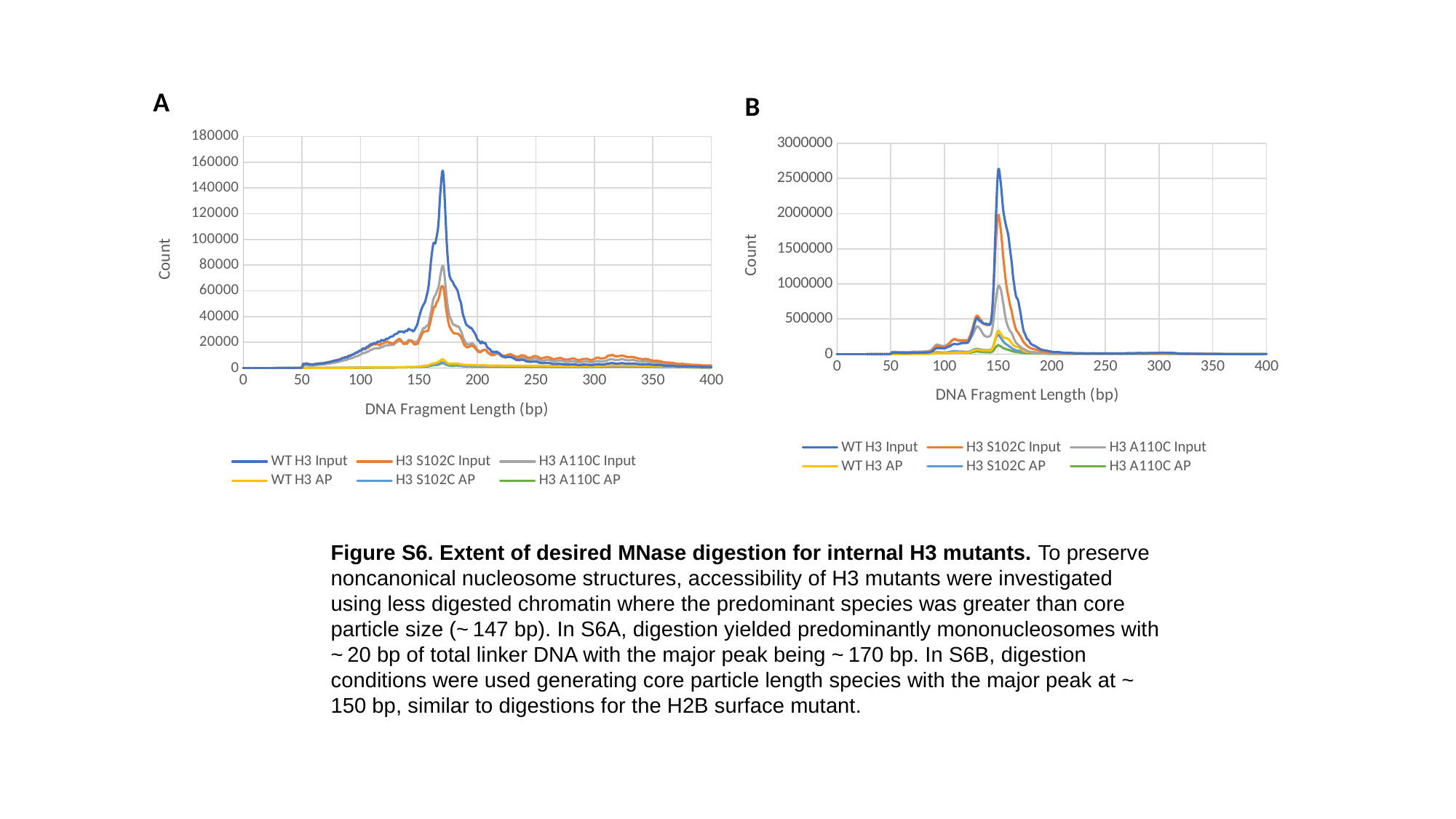

A
B
### Chart
| Category | WT H3 Input | H3 S102C Input | H3 A110C Input | WT H3 AP | H3 S102C AP | H3 A110C AP |
|---|---|---|---|---|---|---|
### Chart
| Category | WT H3 Input | H3 S102C Input | H3 A110C Input | WT H3 AP | H3 S102C AP | H3 A110C AP |
|---|---|---|---|---|---|---|Figure S6. Extent of desired MNase digestion for internal H3 mutants. To preserve noncanonical nucleosome structures, accessibility of H3 mutants were investigated using less digested chromatin where the predominant species was greater than core particle size (~ 147 bp). In S6A, digestion yielded predominantly mononucleosomes with ~ 20 bp of total linker DNA with the major peak being ~ 170 bp. In S6B, digestion conditions were used generating core particle length species with the major peak at ~ 150 bp, similar to digestions for the H2B surface mutant.

## Slide 8
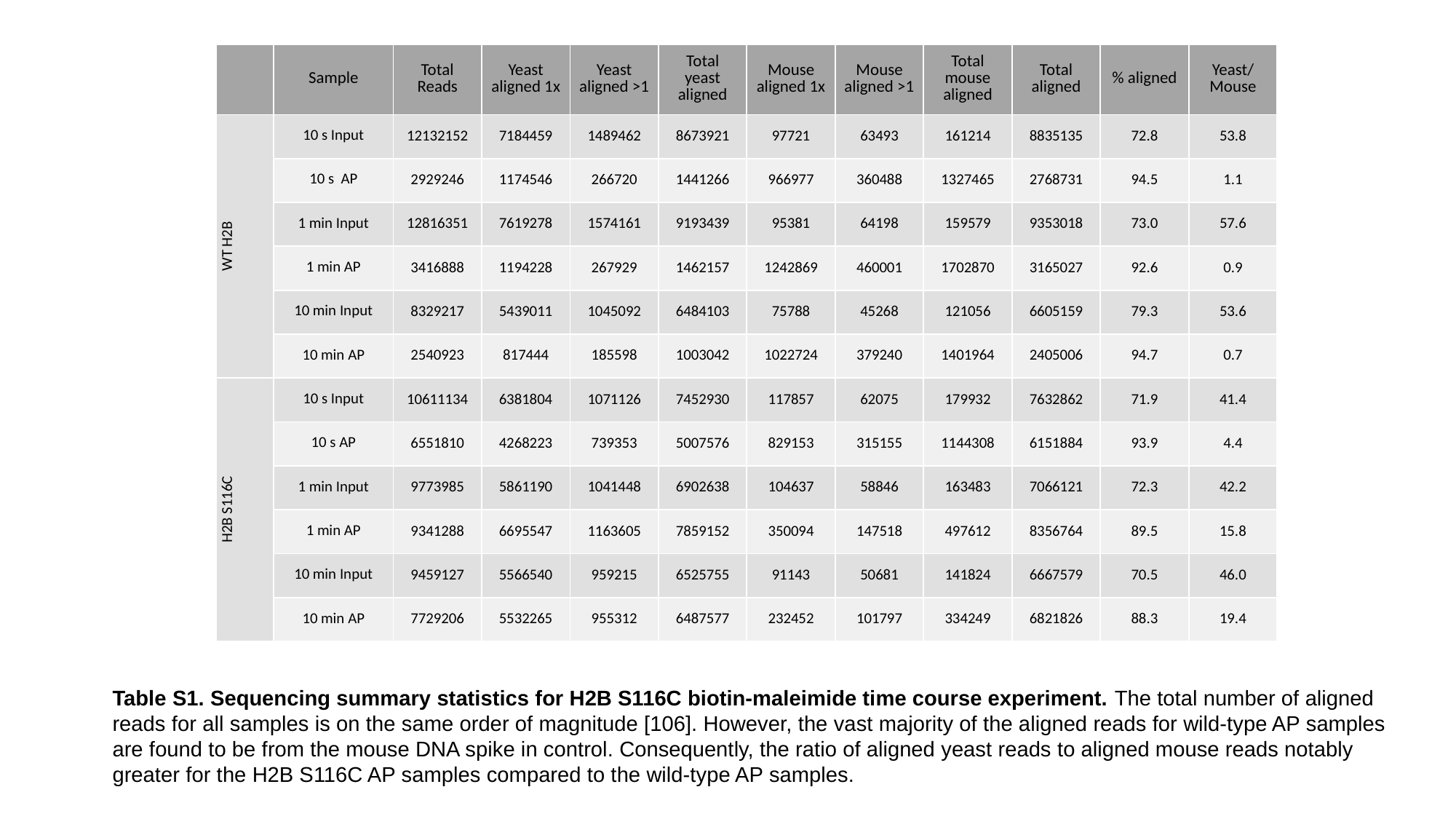

| | Sample | Total Reads | Yeast aligned 1x | Yeast aligned >1 | Total yeast aligned | Mouse aligned 1x | Mouse aligned >1 | Total mouse aligned | Total aligned | % aligned | Yeast/Mouse |
| --- | --- | --- | --- | --- | --- | --- | --- | --- | --- | --- | --- |
| WT H2B | 10 s Input | 12132152 | 7184459 | 1489462 | 8673921 | 97721 | 63493 | 161214 | 8835135 | 72.8 | 53.8 |
| | 10 s AP | 2929246 | 1174546 | 266720 | 1441266 | 966977 | 360488 | 1327465 | 2768731 | 94.5 | 1.1 |
| | 1 min Input | 12816351 | 7619278 | 1574161 | 9193439 | 95381 | 64198 | 159579 | 9353018 | 73.0 | 57.6 |
| | 1 min AP | 3416888 | 1194228 | 267929 | 1462157 | 1242869 | 460001 | 1702870 | 3165027 | 92.6 | 0.9 |
| | 10 min Input | 8329217 | 5439011 | 1045092 | 6484103 | 75788 | 45268 | 121056 | 6605159 | 79.3 | 53.6 |
| | 10 min AP | 2540923 | 817444 | 185598 | 1003042 | 1022724 | 379240 | 1401964 | 2405006 | 94.7 | 0.7 |
| H2B S116C | 10 s Input | 10611134 | 6381804 | 1071126 | 7452930 | 117857 | 62075 | 179932 | 7632862 | 71.9 | 41.4 |
| | 10 s AP | 6551810 | 4268223 | 739353 | 5007576 | 829153 | 315155 | 1144308 | 6151884 | 93.9 | 4.4 |
| | 1 min Input | 9773985 | 5861190 | 1041448 | 6902638 | 104637 | 58846 | 163483 | 7066121 | 72.3 | 42.2 |
| | 1 min AP | 9341288 | 6695547 | 1163605 | 7859152 | 350094 | 147518 | 497612 | 8356764 | 89.5 | 15.8 |
| | 10 min Input | 9459127 | 5566540 | 959215 | 6525755 | 91143 | 50681 | 141824 | 6667579 | 70.5 | 46.0 |
| | 10 min AP | 7729206 | 5532265 | 955312 | 6487577 | 232452 | 101797 | 334249 | 6821826 | 88.3 | 19.4 |
Table S1. Sequencing summary statistics for H2B S116C biotin-maleimide time course experiment. The total number of aligned reads for all samples is on the same order of magnitude [106]. However, the vast majority of the aligned reads for wild-type AP samples are found to be from the mouse DNA spike in control. Consequently, the ratio of aligned yeast reads to aligned mouse reads notably greater for the H2B S116C AP samples compared to the wild-type AP samples.

## Slide 9
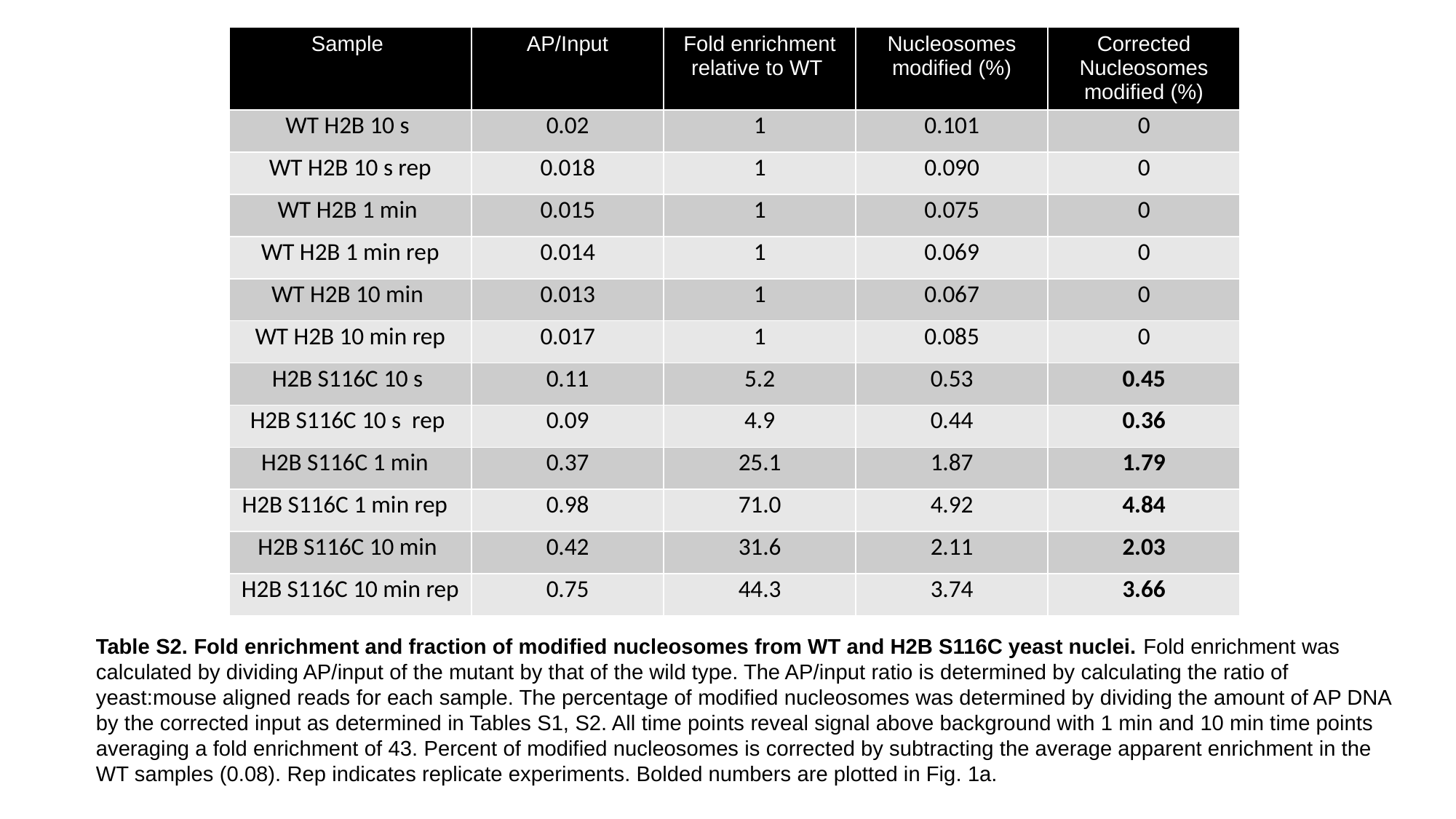

| Sample | AP/Input | Fold enrichment relative to WT | Nucleosomes modified (%) | Corrected Nucleosomes modified (%) |
| --- | --- | --- | --- | --- |
| WT H2B 10 s | 0.02 | 1 | 0.101 | 0 |
| WT H2B 10 s rep | 0.018 | 1 | 0.090 | 0 |
| WT H2B 1 min | 0.015 | 1 | 0.075 | 0 |
| WT H2B 1 min rep | 0.014 | 1 | 0.069 | 0 |
| WT H2B 10 min | 0.013 | 1 | 0.067 | 0 |
| WT H2B 10 min rep | 0.017 | 1 | 0.085 | 0 |
| H2B S116C 10 s | 0.11 | 5.2 | 0.53 | 0.45 |
| H2B S116C 10 s rep | 0.09 | 4.9 | 0.44 | 0.36 |
| H2B S116C 1 min | 0.37 | 25.1 | 1.87 | 1.79 |
| H2B S116C 1 min rep | 0.98 | 71.0 | 4.92 | 4.84 |
| H2B S116C 10 min | 0.42 | 31.6 | 2.11 | 2.03 |
| H2B S116C 10 min rep | 0.75 | 44.3 | 3.74 | 3.66 |
Table S2. Fold enrichment and fraction of modified nucleosomes from WT and H2B S116C yeast nuclei. Fold enrichment was calculated by dividing AP/input of the mutant by that of the wild type. The AP/input ratio is determined by calculating the ratio of yeast:mouse aligned reads for each sample. The percentage of modified nucleosomes was determined by dividing the amount of AP DNA by the corrected input as determined in Tables S1, S2. All time points reveal signal above background with 1 min and 10 min time points averaging a fold enrichment of 43. Percent of modified nucleosomes is corrected by subtracting the average apparent enrichment in the WT samples (0.08). Rep indicates replicate experiments. Bolded numbers are plotted in Fig. 1a.

## Slide 10
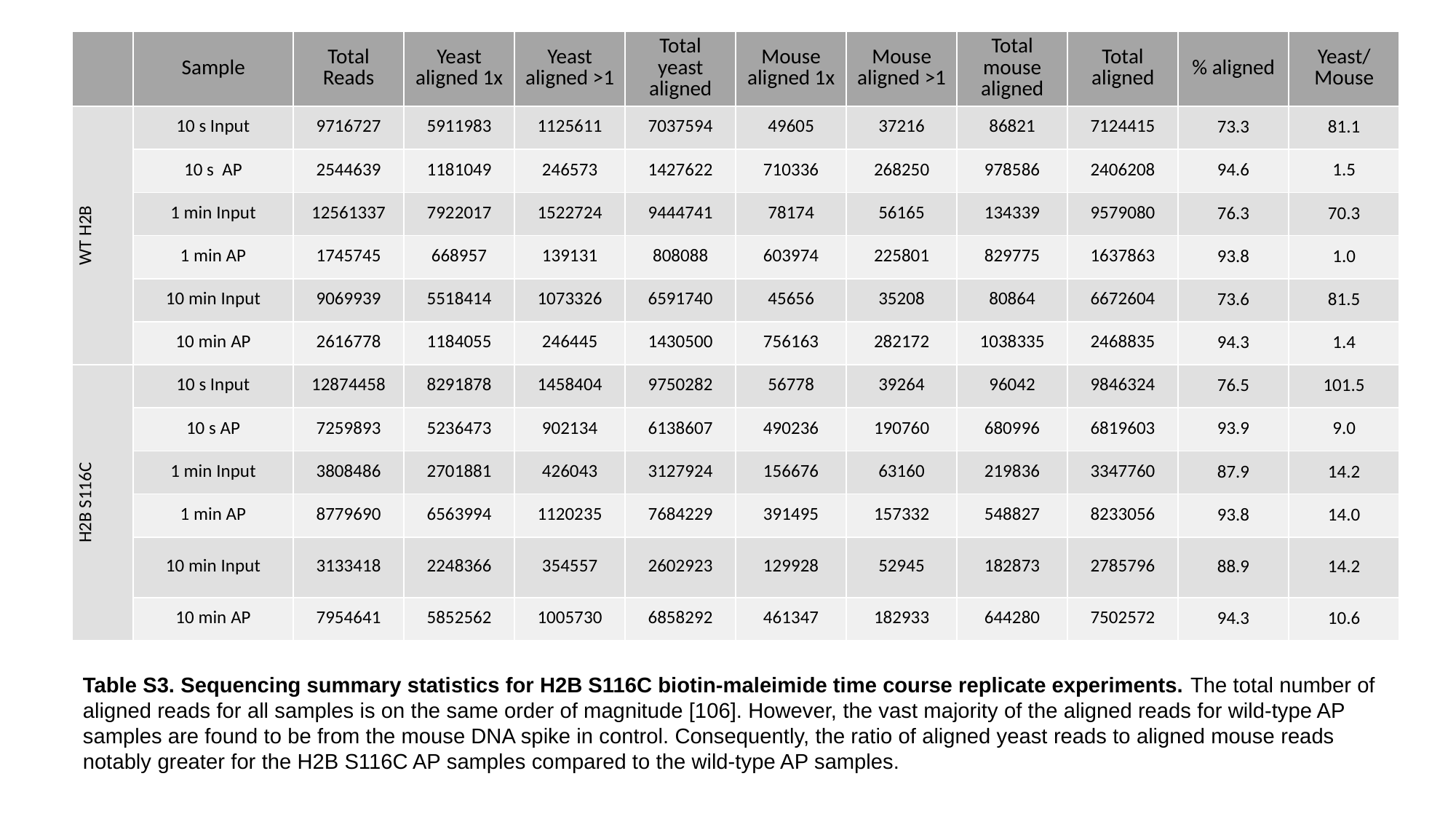

| | Sample | Total Reads | Yeast aligned 1x | Yeast aligned >1 | Total yeast aligned | Mouse aligned 1x | Mouse aligned >1 | Total mouse aligned | Total aligned | % aligned | Yeast/Mouse |
| --- | --- | --- | --- | --- | --- | --- | --- | --- | --- | --- | --- |
| WT H2B | 10 s Input | 9716727 | 5911983 | 1125611 | 7037594 | 49605 | 37216 | 86821 | 7124415 | 73.3 | 81.1 |
| | 10 s AP | 2544639 | 1181049 | 246573 | 1427622 | 710336 | 268250 | 978586 | 2406208 | 94.6 | 1.5 |
| | 1 min Input | 12561337 | 7922017 | 1522724 | 9444741 | 78174 | 56165 | 134339 | 9579080 | 76.3 | 70.3 |
| | 1 min AP | 1745745 | 668957 | 139131 | 808088 | 603974 | 225801 | 829775 | 1637863 | 93.8 | 1.0 |
| | 10 min Input | 9069939 | 5518414 | 1073326 | 6591740 | 45656 | 35208 | 80864 | 6672604 | 73.6 | 81.5 |
| | 10 min AP | 2616778 | 1184055 | 246445 | 1430500 | 756163 | 282172 | 1038335 | 2468835 | 94.3 | 1.4 |
| H2B S116C | 10 s Input | 12874458 | 8291878 | 1458404 | 9750282 | 56778 | 39264 | 96042 | 9846324 | 76.5 | 101.5 |
| | 10 s AP | 7259893 | 5236473 | 902134 | 6138607 | 490236 | 190760 | 680996 | 6819603 | 93.9 | 9.0 |
| | 1 min Input | 3808486 | 2701881 | 426043 | 3127924 | 156676 | 63160 | 219836 | 3347760 | 87.9 | 14.2 |
| | 1 min AP | 8779690 | 6563994 | 1120235 | 7684229 | 391495 | 157332 | 548827 | 8233056 | 93.8 | 14.0 |
| | 10 min Input | 3133418 | 2248366 | 354557 | 2602923 | 129928 | 52945 | 182873 | 2785796 | 88.9 | 14.2 |
| | 10 min AP | 7954641 | 5852562 | 1005730 | 6858292 | 461347 | 182933 | 644280 | 7502572 | 94.3 | 10.6 |
Table S3. Sequencing summary statistics for H2B S116C biotin-maleimide time course replicate experiments. The total number of aligned reads for all samples is on the same order of magnitude [106]. However, the vast majority of the aligned reads for wild-type AP samples are found to be from the mouse DNA spike in control. Consequently, the ratio of aligned yeast reads to aligned mouse reads notably greater for the H2B S116C AP samples compared to the wild-type AP samples.

## Slide 11
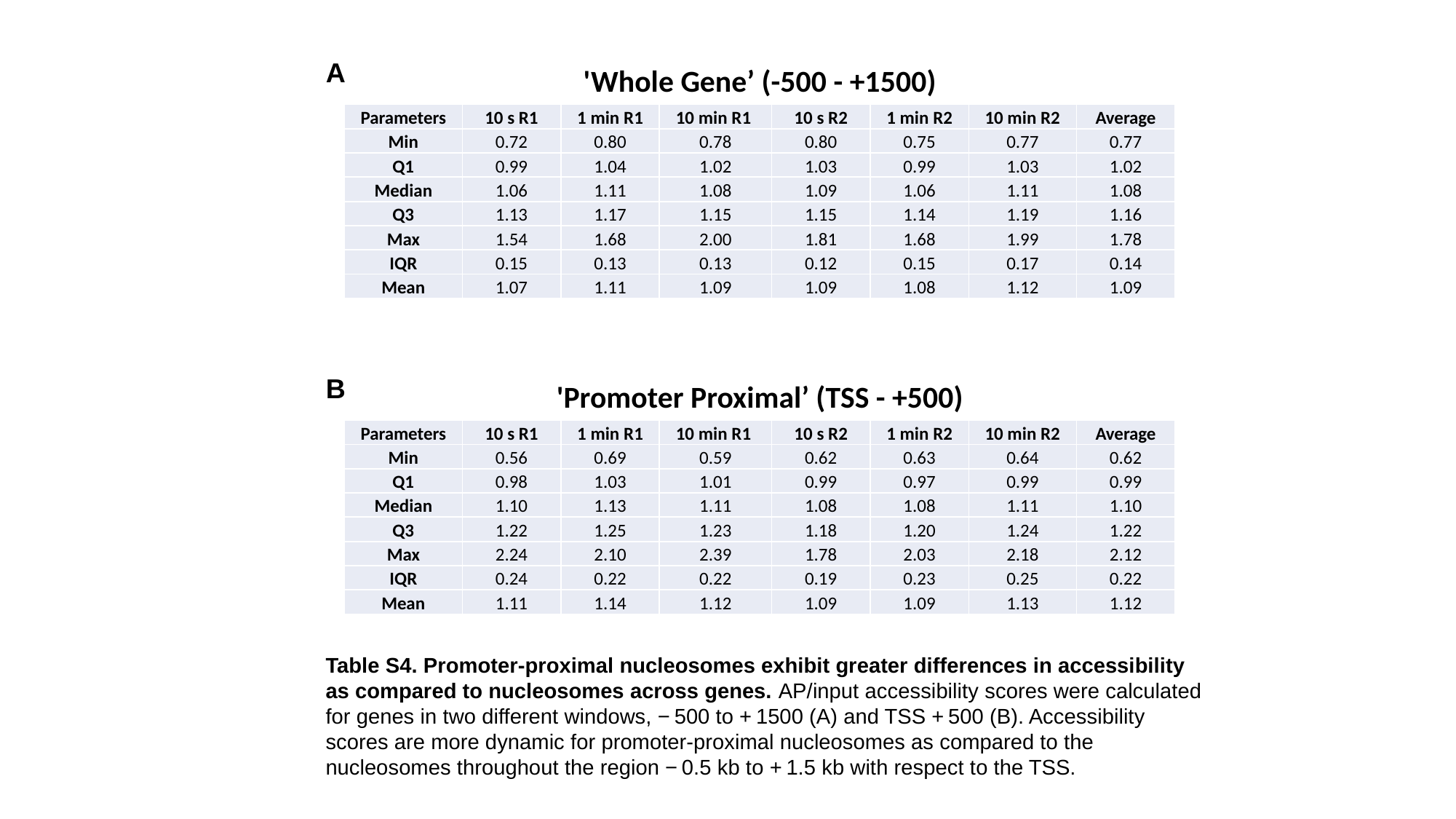

A
'Whole Gene’ (-500 - +1500)
| Parameters | 10 s R1 | 1 min R1 | 10 min R1 | 10 s R2 | 1 min R2 | 10 min R2 | Average |
| --- | --- | --- | --- | --- | --- | --- | --- |
| Min | 0.72 | 0.80 | 0.78 | 0.80 | 0.75 | 0.77 | 0.77 |
| Q1 | 0.99 | 1.04 | 1.02 | 1.03 | 0.99 | 1.03 | 1.02 |
| Median | 1.06 | 1.11 | 1.08 | 1.09 | 1.06 | 1.11 | 1.08 |
| Q3 | 1.13 | 1.17 | 1.15 | 1.15 | 1.14 | 1.19 | 1.16 |
| Max | 1.54 | 1.68 | 2.00 | 1.81 | 1.68 | 1.99 | 1.78 |
| IQR | 0.15 | 0.13 | 0.13 | 0.12 | 0.15 | 0.17 | 0.14 |
| Mean | 1.07 | 1.11 | 1.09 | 1.09 | 1.08 | 1.12 | 1.09 |
B
'Promoter Proximal’ (TSS - +500)
| Parameters | 10 s R1 | 1 min R1 | 10 min R1 | 10 s R2 | 1 min R2 | 10 min R2 | Average |
| --- | --- | --- | --- | --- | --- | --- | --- |
| Min | 0.56 | 0.69 | 0.59 | 0.62 | 0.63 | 0.64 | 0.62 |
| Q1 | 0.98 | 1.03 | 1.01 | 0.99 | 0.97 | 0.99 | 0.99 |
| Median | 1.10 | 1.13 | 1.11 | 1.08 | 1.08 | 1.11 | 1.10 |
| Q3 | 1.22 | 1.25 | 1.23 | 1.18 | 1.20 | 1.24 | 1.22 |
| Max | 2.24 | 2.10 | 2.39 | 1.78 | 2.03 | 2.18 | 2.12 |
| IQR | 0.24 | 0.22 | 0.22 | 0.19 | 0.23 | 0.25 | 0.22 |
| Mean | 1.11 | 1.14 | 1.12 | 1.09 | 1.09 | 1.13 | 1.12 |
Table S4. Promoter-proximal nucleosomes exhibit greater differences in accessibility as compared to nucleosomes across genes. AP/input accessibility scores were calculated for genes in two different windows, − 500 to + 1500 (A) and TSS + 500 (B). Accessibility scores are more dynamic for promoter-proximal nucleosomes as compared to the nucleosomes throughout the region − 0.5 kb to + 1.5 kb with respect to the TSS.

## Slide 12
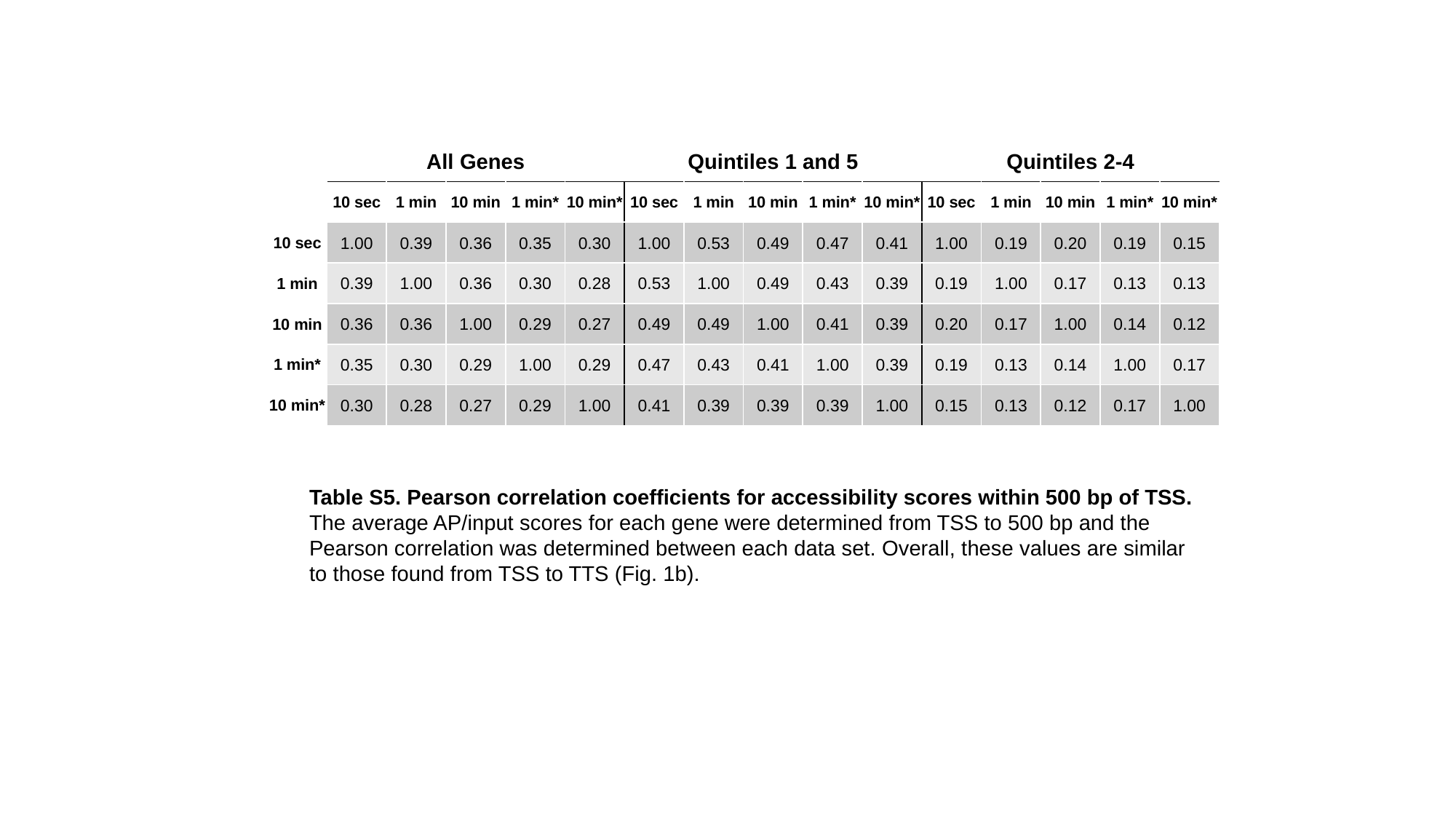

| | All Genes | | | | | Quintiles 1 and 5 | | | | | Quintiles 2-4 | | | | |
| --- | --- | --- | --- | --- | --- | --- | --- | --- | --- | --- | --- | --- | --- | --- | --- |
| | 10 sec | 1 min | 10 min | 1 min\* | 10 min\* | 10 sec | 1 min | 10 min | 1 min\* | 10 min\* | 10 sec | 1 min | 10 min | 1 min\* | 10 min\* |
| 10 sec | 1.00 | 0.39 | 0.36 | 0.35 | 0.30 | 1.00 | 0.53 | 0.49 | 0.47 | 0.41 | 1.00 | 0.19 | 0.20 | 0.19 | 0.15 |
| 1 min | 0.39 | 1.00 | 0.36 | 0.30 | 0.28 | 0.53 | 1.00 | 0.49 | 0.43 | 0.39 | 0.19 | 1.00 | 0.17 | 0.13 | 0.13 |
| 10 min | 0.36 | 0.36 | 1.00 | 0.29 | 0.27 | 0.49 | 0.49 | 1.00 | 0.41 | 0.39 | 0.20 | 0.17 | 1.00 | 0.14 | 0.12 |
| 1 min\* | 0.35 | 0.30 | 0.29 | 1.00 | 0.29 | 0.47 | 0.43 | 0.41 | 1.00 | 0.39 | 0.19 | 0.13 | 0.14 | 1.00 | 0.17 |
| 10 min\* | 0.30 | 0.28 | 0.27 | 0.29 | 1.00 | 0.41 | 0.39 | 0.39 | 0.39 | 1.00 | 0.15 | 0.13 | 0.12 | 0.17 | 1.00 |
Table S5. Pearson correlation coefficients for accessibility scores within 500 bp of TSS. The average AP/input scores for each gene were determined from TSS to 500 bp and the Pearson correlation was determined between each data set. Overall, these values are similar to those found from TSS to TTS (Fig. 1b).

## Slide 13
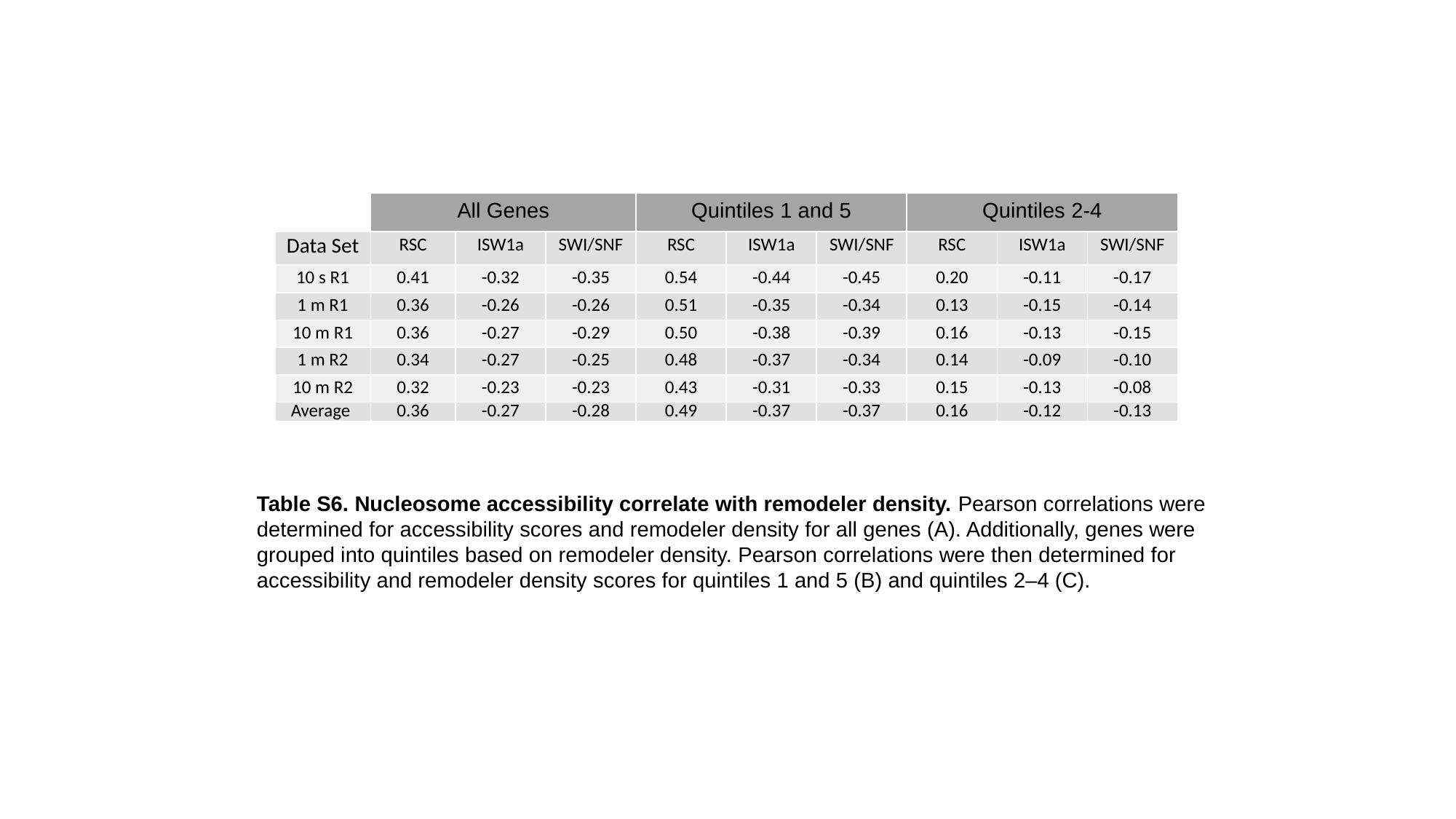

| | All Genes | | | Quintiles 1 and 5 | | | Quintiles 2-4 | | |
| --- | --- | --- | --- | --- | --- | --- | --- | --- | --- |
| Data Set | RSC | ISW1a | SWI/SNF | RSC | ISW1a | SWI/SNF | RSC | ISW1a | SWI/SNF |
| 10 s R1 | 0.41 | -0.32 | -0.35 | 0.54 | -0.44 | -0.45 | 0.20 | -0.11 | -0.17 |
| 1 m R1 | 0.36 | -0.26 | -0.26 | 0.51 | -0.35 | -0.34 | 0.13 | -0.15 | -0.14 |
| 10 m R1 | 0.36 | -0.27 | -0.29 | 0.50 | -0.38 | -0.39 | 0.16 | -0.13 | -0.15 |
| 1 m R2 | 0.34 | -0.27 | -0.25 | 0.48 | -0.37 | -0.34 | 0.14 | -0.09 | -0.10 |
| 10 m R2 | 0.32 | -0.23 | -0.23 | 0.43 | -0.31 | -0.33 | 0.15 | -0.13 | -0.08 |
| Average | 0.36 | -0.27 | -0.28 | 0.49 | -0.37 | -0.37 | 0.16 | -0.12 | -0.13 |
Table S6. Nucleosome accessibility correlate with remodeler density. Pearson correlations were determined for accessibility scores and remodeler density for all genes (A). Additionally, genes were grouped into quintiles based on remodeler density. Pearson correlations were then determined for accessibility and remodeler density scores for quintiles 1 and 5 (B) and quintiles 2–4 (C).

## Slide 14
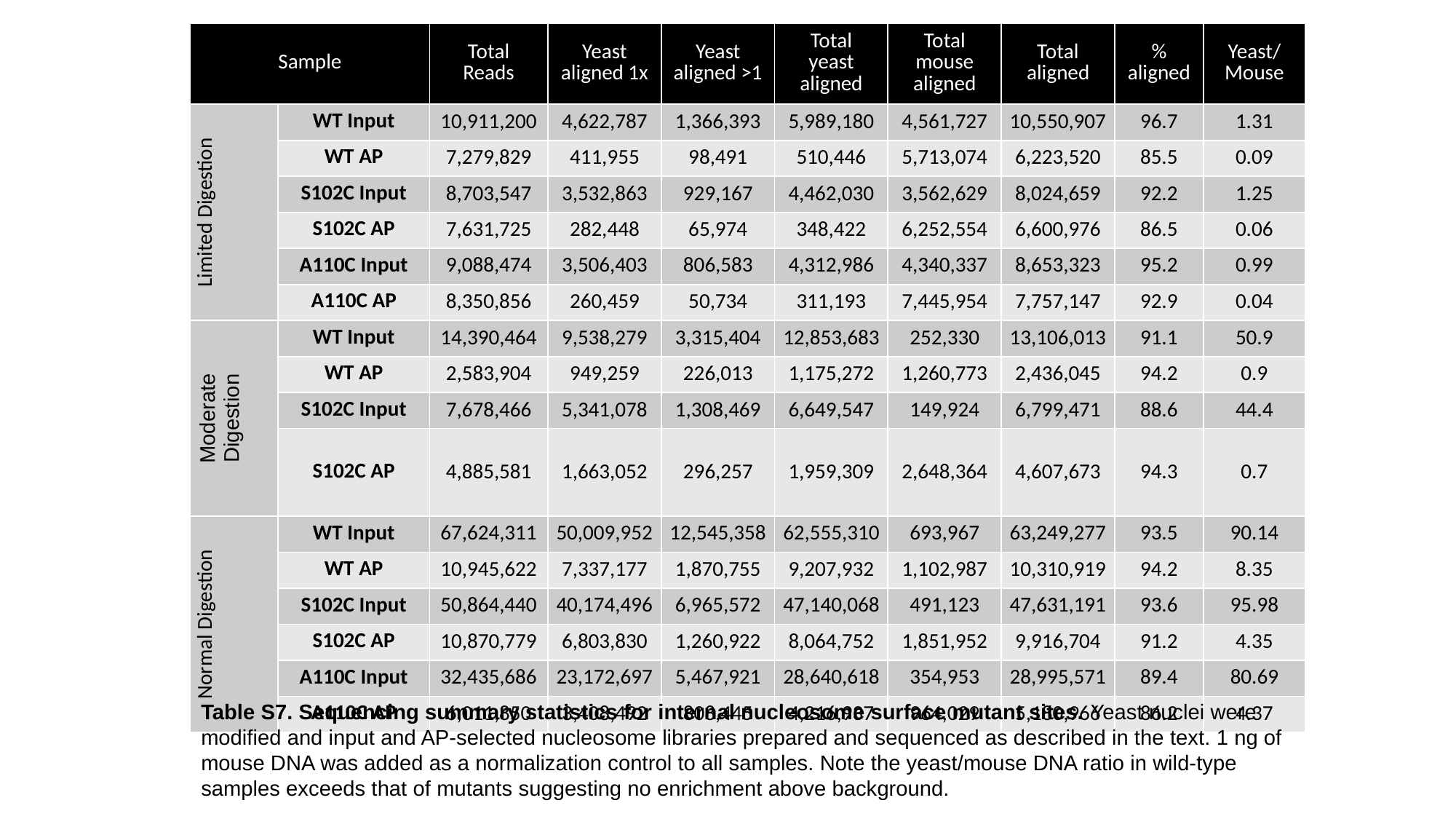

| Sample | | Total Reads | Yeast aligned 1x | Yeast aligned >1 | Total yeast aligned | Total mouse aligned | Total aligned | % aligned | Yeast/Mouse |
| --- | --- | --- | --- | --- | --- | --- | --- | --- | --- |
| Limited Digestion | WT Input | 10,911,200 | 4,622,787 | 1,366,393 | 5,989,180 | 4,561,727 | 10,550,907 | 96.7 | 1.31 |
| | WT AP | 7,279,829 | 411,955 | 98,491 | 510,446 | 5,713,074 | 6,223,520 | 85.5 | 0.09 |
| | S102C Input | 8,703,547 | 3,532,863 | 929,167 | 4,462,030 | 3,562,629 | 8,024,659 | 92.2 | 1.25 |
| | S102C AP | 7,631,725 | 282,448 | 65,974 | 348,422 | 6,252,554 | 6,600,976 | 86.5 | 0.06 |
| | A110C Input | 9,088,474 | 3,506,403 | 806,583 | 4,312,986 | 4,340,337 | 8,653,323 | 95.2 | 0.99 |
| | A110C AP | 8,350,856 | 260,459 | 50,734 | 311,193 | 7,445,954 | 7,757,147 | 92.9 | 0.04 |
| Moderate Digestion | WT Input | 14,390,464 | 9,538,279 | 3,315,404 | 12,853,683 | 252,330 | 13,106,013 | 91.1 | 50.9 |
| | WT AP | 2,583,904 | 949,259 | 226,013 | 1,175,272 | 1,260,773 | 2,436,045 | 94.2 | 0.9 |
| | S102C Input | 7,678,466 | 5,341,078 | 1,308,469 | 6,649,547 | 149,924 | 6,799,471 | 88.6 | 44.4 |
| | S102C AP | 4,885,581 | 1,663,052 | 296,257 | 1,959,309 | 2,648,364 | 4,607,673 | 94.3 | 0.7 |
| Normal Digestion | WT Input | 67,624,311 | 50,009,952 | 12,545,358 | 62,555,310 | 693,967 | 63,249,277 | 93.5 | 90.14 |
| | WT AP | 10,945,622 | 7,337,177 | 1,870,755 | 9,207,932 | 1,102,987 | 10,310,919 | 94.2 | 8.35 |
| | S102C Input | 50,864,440 | 40,174,496 | 6,965,572 | 47,140,068 | 491,123 | 47,631,191 | 93.6 | 95.98 |
| | S102C AP | 10,870,779 | 6,803,830 | 1,260,922 | 8,064,752 | 1,851,952 | 9,916,704 | 91.2 | 4.35 |
| | A110C Input | 32,435,686 | 23,172,697 | 5,467,921 | 28,640,618 | 354,953 | 28,995,571 | 89.4 | 80.69 |
| | A110C AP | 6,011,850 | 3,408,492 | 808,445 | 4,216,937 | 964,029 | 5,180,966 | 86.2 | 4.37 |
Table S7. Sequencing summary statistics for internal nucleosome surface mutant sites. Yeast nuclei were modified and input and AP-selected nucleosome libraries prepared and sequenced as described in the text. 1 ng of mouse DNA was added as a normalization control to all samples. Note the yeast/mouse DNA ratio in wild-type samples exceeds that of mutants suggesting no enrichment above background.
